# Temperature-pressure phase diagram of confined monolayer water/ice at first-principles accuracy with a machine-learning force field

**DOI:** 10.1038/s41467-023-39829-z

**Published:** 2023-07-11

**Authors:** Bo Lin, Jian Jiang, Xiao Cheng Zeng, Lei Li

**Affiliations:** 1 Guangdong Provincial Key Laboratory of Functional Oxide Materials and Devices, Department of Materials Science and Engineering, Southern University of Science and Technology, Shenzhen, Guangdong, 518055 China; 2 Department of Materials Science and Engineering, City University of Hong Kong, Kowloon, 999077 Hong Kong; 3 Department of Chemistry, University of Nebraska-Lincoln, Lincoln, NE 68588 USA

**Keywords:** Structure prediction, Phase transitions and critical phenomena

## Abstract

Understanding the phase behaviour of nanoconfined water films is of fundamental importance in broad fields of science and engineering. However, the phase behaviour of the thinnest water film – monolayer water – is still incompletely known. Here, we developed a machine-learning force field (MLFF) at first-principles accuracy to determine the phase diagram of monolayer water/ice in nanoconfinement with hydrophobic walls. We observed the spontaneous formation of two previously unreported high-density ices, namely, zigzag quasi-bilayer ice (ZZ-qBI) and branched-zigzag quasi-bilayer ice (bZZ-qBI). Unlike conventional bilayer ices, few inter-layer hydrogen bonds were observed in both quasi-bilayer ices. Notably, the bZZ-qBI entails a unique hydrogen-bonding network that consists of two distinctive types of hydrogen bonds. Moreover, we identified, for the first time, the stable region for the lowest-density $4\cdot {8}^{2}$ monolayer ice (LD-48MI) at negative pressures (<−0.3 GPa). Overall, the MLFF enables large-scale first-principle-level molecular dynamics (MD) simulations of the spontaneous transition from the liquid water to a plethora of monolayer ices, including hexagonal, pentagonal, square, zigzag (ZZMI), and hexatic monolayer ices. These findings will enrich our understanding of the phase behaviour of the nanoconfined water/ices and provide a guide for future experimental realization of the 2D ices.

## Abstract (web summary)

Understanding the phase behaviour of nanoconfined water is of importance in science & engineering. Here the authors use machine-learning force field molecular dynamics to report two new quasi-bilayer ice phases in the phase diagram of monolayer ices.

## Introduction

Water and ice in nanoscale confinement have attracted intense attention due to their fundamental relevance to many scientific disciplines, including environmental science^1–5^, condensed matter physics^6–11^, cell biology^12,13^ and nanoscience^14–16^. The nano-confined two-dimensional (2D) water exhibits distinctly different phase behaviour from its bulk counterparts. For example, the 2D ice confined in graphene nanocapillaries can exhibit a square-lattice structure, as detected from the transmission electron microscope (TEM) measurement^7^, in stark contrast to the diamond-like structure^17^ of bulk ice I. Also, unlike bulk ice I – XVII, the theoretically predicted monolayer ice^18,19^ may not necessarily follow the bulk ice rule^20^ (that is, each water molecule forms four hydrogen bonds with four nearest neighbouring water molecules). In addition, nano-confined 2D ices can also exhibit distinctive dynamical and mechanical properties^3,8^ unseen in bulk ices, and these properties are highly affected by the confinement dimensions, in addition to the temperature and pressure. An improved understanding of the phase behaviour of 2D water/ice in nano-confinement will have important implication for many related applications such as nanofluidic, interface chemistry and low-dimensional physics.

To date, many crystalline structures of 2D ices have been identified from molecular dynamics (MD) simulations, for example, square-octagonal^18,21,22^, hexagonal^22–26^, pentagonal^18,27,28^, square^23,25,29,30^, planar rhombic^18,22,30,31^ and puckered rhombic^18,19,22,31^ structures. Recent experiments have also observed the formation of 2D square ice^7^ within graphene nanocapillaries and bilayer hexagonal ices^1^ on Au(111) surface. The square-octagonal and hexagonal monolayer ices have an area density of 9 and 11 nm^−2^, respectively, and thus are viewed as low- and mid-density ices^18^. The square-octagonal ice is also known as the low-density $4\cdot {8}^{2}$ monolayer ice (LD-48MI)^21^. Other 2D ices have an area density > 12 nm^−2^ and thus belong to high-density ices. Most of these 2D ices are obtained based on MD simulations^18,32–35^ with different classical force fields of rigid water, such as TIP4P^36,37^, TIP5P^38^ and SPC/E^39^. Thus far, only the formation of the rhombic and square-octagonal monolayer ices has been observed to undergo a 2D liquid-to-solid transition^21,22^, while others generally involve a solid-to-solid transition.

Another important issue^30,40^ is the dependence of the predicted ice structures on the selected classical force field (FF). For example, the coffin bilayer ice obtained with the 4-site TIP4P water model was not seen in MD simulations with the 5-site TIP5P model. Likewise, the interlocked pentagonal bilayer ice obtained in MD simulations^28^ with the TIP5P model was not seen with the TIP4P model. Note that most classical water models adopt rigid oxygen-hydrogen (O − H) bonds and rigid H − O − H angles with little polarizability^36–39^. These approximations to the realistic water molecules could overlook certain regions of the phase space and associated ice structures. For instance, MD simulations with the SPC/E water model^18^ predicted a near-rhomboidal monolayer structure rather than the square monolayer structure as detected in the TEM experiment^7^.

In addition to classical MD simulations, first-principles density functional theory (DFT) calculations and ab initio MD (AIMD) simulations have been used to explore the phase behaviour of monolayer ice. Chen et al.^19^ employed a random structure search method based on DFT calculation and investigated the relative stability of four monolayer ices, i.e., hexagonal, pentagonal Cairo-tiled, flat square, and buckled rhombic monolayer ices. Jiang et al.^20^ observed the spontaneous freezing transition of the 2D water to the zigzag monolayer ice (ZZMI) and to the bilayer *ice*-VII-like ice (BL-*i*VII) in their AIMD simulations. Compared to classical MD simulations, AIMD simulations with first-principles accuracy are much more reliable in predicting the structure and stability of ices. However, due to the high computational cost, AIMD simulations are often limited to ~1–2 nm in spatial dimension and tens of picoseconds in timescale, thereby rendering the phase transition that requires timescale beyond nanoseconds or length scale beyond 3 nm computationally impractical.

Recently, the machine-learning force field (MLFF) has shown the emerging possibility to narrow the gap between the first-principle accuracy and classical-FFs efficiency and is considered a reliable solution to meet the need for more realistic large-scale MD simulations^41–43^. MLFF has been used to simulate the crystal nucleation of silicon^44,45^, gallium^46^ and bulk water^47^. Kapil et al.^48^ coupled such a technique with the thermodynamics integration method to compute the free energy of the monolayer ice. They reported the pressure-temperature phase diagram of the monolayer ice with the lateral pressure ranging from 0 to 5 GPa and temperature ranging from 0 to 600 K. They found various phases of the monolayer ices, including hexagonal, pentagonal, square, flat-rhombic (i.e., flat-ZZMI), and hexatic monolayer, as well as a new superionic monolayer phase. However, the phase behaviour of the monolayer water beyond 0–5 GPa is yet to be investigated. Moreover, simulation evidence for the spontaneous liquid-to-solid and solid-to-solid transition with MLFF would be highly desirable to trace the dynamics of the phase transition.

Understanding the phase behaviour of the 2D water beyond 0–5 GPa range has fundamental implications in physics, chemistry, geoscience, and planetary science. In particular, the phase behaviour of the 2D water at the negative pressure, compared to the positive-pressure region, is much less explored. Water behaviour at negative pressure is expected to be dominated by intermolecular attractive interaction rather than repulsive one. Hence, the underlying physics would be very different from that in the positive-pressure region. Remarkably, the guest-free ice clathrate (i.e., ice XVI) with low density (0.81 g⋅cm^−3^) was recently fabricated in the laboratory, inspiring further investigation of low-density ices at negative pressure^49^. Low-density monolayer ice, LD-48MI, was theoretically predicted to be stable at negative pressure^21^ although the detailed thermodynamic stable region is still little known. Matter under high-pressure conditions is a central subject in planetary science and high-pressure physics, including the topics of the phase and dynamic behaviour of materials at high pressures^50,51^. For example, water trapped in the mantle of Earth is under high pressure up to 24 GPa^52–54^. Jupiter, with typical pressure an order of magnitude higher than Earth, has been proven to have water^55,56^. In laboratory, high pressure beyond 5 GPa can be commonly achieved via the use of diamond anvil cells (the high-end pressure range is typically 100–200 GPa)^57,58^. This experimental technique has been widely employed in studying the phase behaviour of 2D materials^59–65^.

In this work, we developed an MLFF with the DeePMD-kit^42^ package for MD simulations in order to investigate the phase transitions of monolayer water/ice systems confined to a nanoslit with 6.0 Å width. Simulation evidence of the spontaneous solid-to-solid transition and the freezing transition to hexagonal, pentagonal, square, flat-ZZMI and hexatic ices, were obtained via lowering temperature in steps. Moreover, sequential phase transitions from the monolayer liquid to quasi-bilayer ice phases were observed upon increasing lateral pressure in steps. When combined with the thermodynamics-integration method, a more complete phase diagram of the monolayer water/ice in the lateral pressure range of −0.5 GPa ≤ *P* ≤ 20 GPa and *T* ≤ 400 K was achieved. Especially, a stable negative-pressure region for the low-density monolayer ice - LD-48MI–was identified, thereby enriching the state-of-the-art phase diagram of monolayer water/ices.

## Results and discussion

### An MLFF model for the 2D water/ice system

**Fig. 1:**
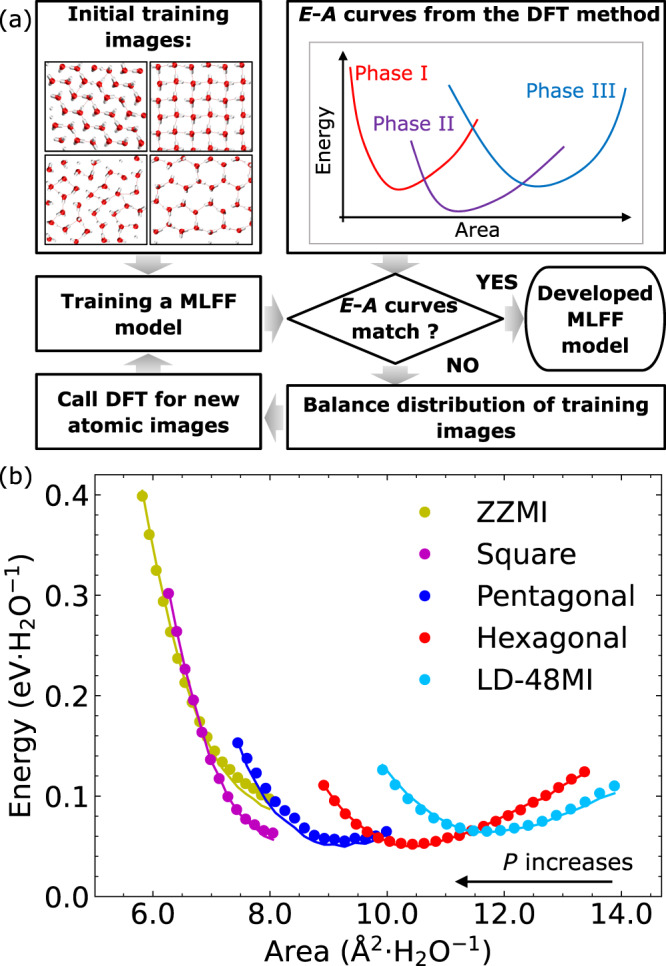
Development of the machine-learning force field (MLFF) model for the 2D water/ice system. **a** Flowchart of the active-learning approach for developing the MLFF model. Initial training images were collected from ref. ^20^ and molecular dynamics (MD) simulations of the five previously reported 2D ices indicated in (**b**). The obtained MLFF model was benchmarked with the energy-area (*E*-*A*) curves of the five known 2D ices. The area density distribution of the training images was evaluated if the *E-A* curves did not match, and new training data were collected with ab initio MD (AIMD) and MLFF-based MD simulations (for temperature range of 10–400 K) to balance the distribution. **b** *E*-*A* curves of five 2D ice phases, calculated with the density functional theory (DFT) method (solid line) and the MLFF model (dots). The energy *E* is defined as the energy per water molecule, and the corresponding area *A* is calculated with $A={L}_{x}\times {L}_{y}/{N}_{{{{{{\rm{water}}}}}}}$. *L*_*x*_ and *L*_*y*_ are lengths of the simulation box along the *x*- and *y*-axis, respectively. ZZMI and LD-48MI represent the zigzag monolayer ice and low-density 4⋅8^2^ monolayer ice, respectively. Source data are provided as a Source Data file^78^.

First, we trained an MLFF model for the monolayer 2D water/ices with the van der Waals density functional (vdW-DF2) as the reference. The vdW-DF2 functional is among the best DFT functions to describe the 2D water/ice system when compared with the quantum Monte Carlo method, as shown in Table 4 (ref. ^66^) where columns 4 and 5 summarized the error of various functionals for the 2D/3D ices^66^. An active learning approach shown in Fig. 1a was employed to obtain the MLFF model that can reproduce the energy-area (*E*-*A*) curves of the five reported 2D ices (see Fig. 1a and the Supplementary Information for more details), including the LD-48MI, hexagonal, pentagonal, square and zig-zag monolayer ices. Like the energy-volume (*E*-*V*) curve in bulk systems, the *E*-*A* curve can be a valuable base to assess the phase behaviour of the 2D water/ice system^67^. Thus, accurate *E*-*A* curve is a crucial step towards more comprehensive simulations of phase behaviours of 2D water/ice systems at finite temperatures.

After the MLFF training, the root-mean-square error (RMSE) of the energy and the force reach ~4.9 meV⋅H_2_O^−1^ and ~78.5 meV⋅Å^−1^, respectively. The predicted energy and forces of the 2D water/ice show a tight linear correlation with the reference values along the *y* = *x* line (see Supplementary Fig. 1). The predicted *E*-*A* curves for the five 2D distinct ices based on the MLFF model (solid dots in Fig. 1b) exhibit excellent agreement with the vdW-DF2 computational results (solid lines in Fig. 1b). It determines the phase stability of the 2D water/ice system at 0 K. At 0 K, among ices with *A* < 10.8 Å^2^, the hexagonal ice is predicted to be the most stable at the low-pressure region. With increasing pressure, the pentagonal, square and zig-zag monolayer ices become the most stable phase in succession. Such a stability trend is consistent with the results reported by Kapil et al.^48^

### MD simulations of spontaneous liquid-to-solid and solid-to-solid transition of the 2D water/ice system

Direct simulation evidences of phase transitions, particularly the freezing process of the liquid phase, are desirable for assessing the dynamic attainability of the 2D ices and assessing the temperature and pressure range for determination of the liquid-solid phase boundary^20^. Here, we performed MD simulations of the freezing dynamics of the 2D water (having 192 water molecules) confined within a 6.0 Å wide nanoslit with the developed MLFF model (see Methods section for more details). During the simulations, the confined water initially stabilized at 320 K underwent a stepwise cooling process, followed by recursive annealing processes in the pressure range of −0.3 to 10 GPa and at *T* ≤ 250 K. Temperature evolution during the annealing processes was shown in Supplementary Fig. 3. The spontaneous formation of the hexagonal, pentagonal and square monolayer ices was observed, respectively. The centro-symmetry parameter (${p}_{{{{{{\rm{CSP}}}}}}}^{N}$) was used to track the crystallization process of the monolayer water. For a water molecule with *N* nearest neighbours, the ${p}_{{{{{{\rm{CSP}}}}}}}^{N}$ is calculated according to below equation:

1 $${p}_{{{{{{\rm{CSP}}}}}}}^{N}=\frac{1}{{N}_{w}}{\sum }_{{{{{{\rm{i}}}}}}=1}^{{N}_{w}}\left( \left|{\sum }_{j-1}^{N}{{{{{{\bf{r}}}}}}}_{{{{{{\bf{ij}}}}}}} \right|\right)$$

where *N*_*w*_ is the total number of water molecules in the system, and **r**_**ij**_ is the vector from the centre water molecule *i* to the neighbour *j*. The evolution of the oxygen-oxygen (O-O) pair distribution (*g*_oo_(*r*)) function with time was also computed to further validate the crystallization process.

**Fig. 2:**
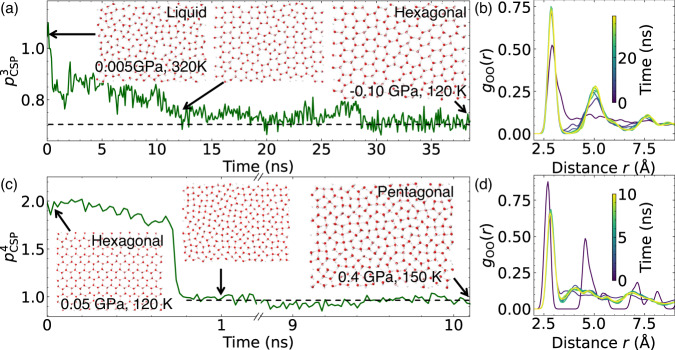
Phase transition processes of the 2D water/ice system as shown from molecular dynamics (MD) simulations. **a** The evolution of the centro-symmetry parameter (CSP) of the water molecule with three nearest oxygen atoms, ${p}_{{{{{{\rm{CSP}}}}}}}^{3}$, and (**b**) the pair distribution function (RDF) of the oxygen atoms, *g*_oo_(*r*), during the MD simulations of spontaneous formation of hexagonal ice from the 2D water. **c** The evolution of ${p}_{{{{{{\rm{CSP}}}}}}}^{4}$, and (**d**) *g*_oo_(*r*), during the MD simulation of the hexagonal-to-pentagonal transition. The white and red spheres represent hydrogen and oxygen atoms, respectively. ${p}_{{{{{{\rm{CSP}}}}}}}^{N}$ is defined by Eq.1. Source data are provided as a Source Data file^78^.

Take the formation of the hexagonal monolayer ice as a typical example. The confined water was first cooled from 320 K to 120 K in steps at constant pressure, and then underwent recursive annealing processes with a temperature in the range of 120 K to 160 K. Figure 2a, b show the variation of the ${p}_{{{{{{\rm{CSP}}}}}}}^{3}$ and *g*_oo_(*r*) during the freezing process. Supplementary Movie 1 describes the freezing transition. Initially, the monolayer water exhibits a relatively large ${p}_{{{{{{\rm{CSP}}}}}}}^{3}$ value of 1.1 due to its disordered structure as characterized by a continuous distribution of the *g*_oo_(*r*) function (dark red line in Fig. 2b). With lowering the temperature, the water molecules started to form high-symmetry hexagonal monolayer structures, leading to a decrease in the ${p}_{{{{{{\rm{CSP}}}}}}}^{3}$ value. During this process, the peaks in the range of 3.5 to 4 Å in *g*_oo_(*r*) gradually vanished due to the crystallization of the 2D water. At 28.5 ns, a near-perfect hexagonal monolayer ice was observed with an average ${p}_{{{{{{\rm{CSP}}}}}}}^{3}$ value of ~0.7, close to that of the perfect hexagonal monolayer ice at ~120 K. The slightly higher ${p}_{{{{{{\rm{CSP}}}}}}}^{3}$ value observed in the MD simulation is due to the existence of local defective structures, such as five-, seven- and eight-member rings. Two separate peaks were observed in the *g*_oo_(*r*) function (blue lines in Fig. 2b), corresponding to the first- and second-nearest neighbours in the hexagonal monolayer ice. Non-continuous distribution of the *g*_oo_(*r*) function suggests the crystallization of the 2D water.

Besides the hexagonal monolayer ice, we also observed the spontaneous formation of the pentagonal, square monolayer ices and ZZMI from the 2D water (Supplementary Movies 2–4). The evolution of the corresponding ${p}_{{{{{{\rm{CSP}}}}}}}^{N}$ and *g*_oo_(*r*) as shown in Supplementary Figs. 4–6 presents their crystallization process from the 2D water. Because of their higher area density than the hexagonal monolayer ice, a much faster freezing transition (in 4 ns) was generally observed for all three monolayer ices. Among them, the ZZMI with the highest area density only took 0.5 ns to reach the crystallization state with little defect. Overall, the direct observation of the freezing transition to the four monolayer ices provides direct molecular-level evidence of their dynamic accessibility, thereby confirming the existence of a phase boundary with the liquid phase.

To further seek possible solid-to-solid transitions, we ran MD simulations with the obtained crystal ices by varying the lateral pressure, and indeed, we observed spontaneous solid-to-solid transitions. Figure 2c, d shows the evolution of the ${p}_{{{{{{\rm{CSP}}}}}}}^{N}$ and the *g*_oo_(*r*) function during the hexagonal-to-pentagonal monolayer ice transition. The corresponding trajectory of the MD simulation is given by Supplementary Movie 5. The main difference between the hexagonal and pentagonal monolayer ices is that each water molecule has only three nearest hydrogen-bonding neighbours in the former, but four hydrogen-bonding neighbours in the latter. Thus, the ${p}_{{{{{{\rm{CSP}}}}}}}^{4}$ was computed to track the solid-to-solid transition from the hexagonal to the pentagonal monolayer ice. As shown in Fig. 2c, a sudden drop of ${p}_{{{{{{\rm{CSP}}}}}}}^{4}$ from ~1.8 to ~1.0 was observed at 0.8 ns, a typical feature of the first-order transition^26,27,35^. During this transition, the ${p}_{{{{{{\rm{CSP}}}}}}}^{4}$ fluctuated around 0.85, an average value of ${p}_{{{{{{\rm{CSP}}}}}}}^{4}$ for the pentagonal ice at 150 K, suggesting the formation of the pentagonal monolayer ice. The evolution of the *g*_oo_(*r*) function also indicates a structure transition from a highly symmetrical hexagonal monolayer structure to a less-symmetrical pentagonal monolayer structure. Besides, the spontaneous pentagonal-to-square and square-to-ZZMI transitions were also observed, as shown in Supplementary Figs. 7, 8, respectively. These three solid-to-solid transitions involve increasing the water area density and are considered the low-to-high area density phase transition, accompanied by lateral pressure increase. In contrast, a transition from high-area density to a low-area density phase requires a decrease in the pressure as shown by the square-to-pentagonal monolayer ice transition (see Supplementary Fig. 9). Corresponding trajectories of these solid-to-solid phase transitions are available in Supplementary Movies 6–8.

### Quasi-bilayer ices at high lateral pressure

**Fig. 3:**
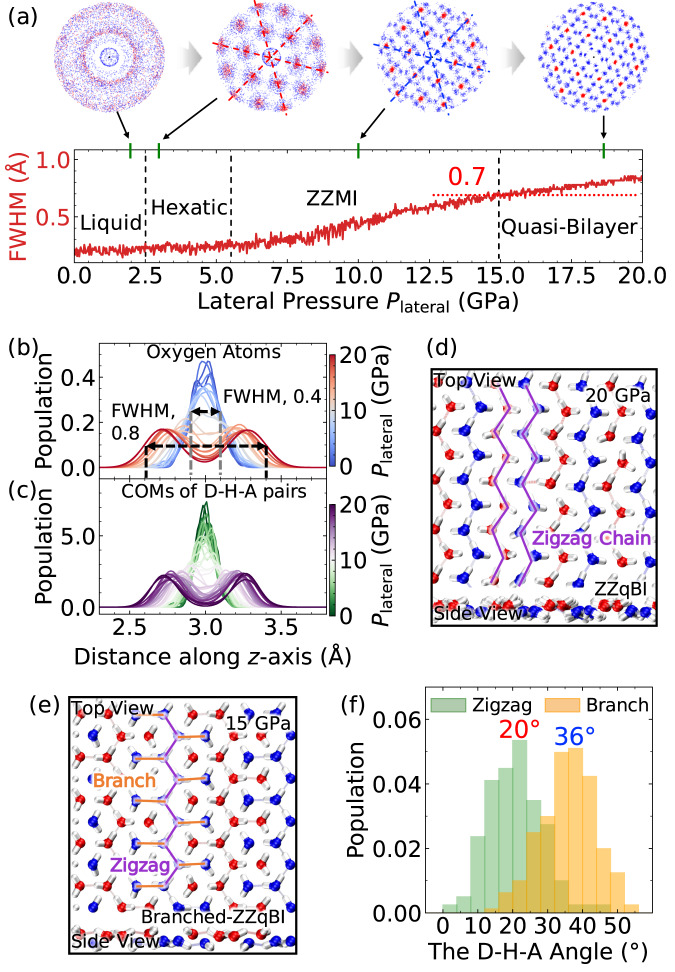
New ice phases observed in molecular dynamics (MD) simulations of sequential phase transition of the 2D water at 300 K. **a** The upper panel displays spatial distributions of oxygen (red dots) and hydrogen (blue dots) within a cutoff of 6 Å at different time slots of the MD simulation. The lower panel shows the variation of full width at half maximum (FWHM, red curves) of the distribution function defined in (**b**). The distribution functions of (**b**) oxygen atoms and (**c**) centre-of-mass (COM) of donor-hydrogen-acceptor (D-H-A) pairs along the *z*-axis evolve with the lateral pressure. **d**, **e** present atomic structures of the zigzag quasi-bilayer ice (ZZ-qBI) and branched-ZZ-qBI (bZZ-qBI), respectively. The white spheres are hydrogen atoms. The red and blue spheres are oxygen atoms in the upper and lower sublayers. **f** Distribution of D-H-A angles (see Supplementary Fig. 15 for the definition) for hydrogen bonds in the zigzag chain (purple lines in (**e**)) and the branches (orange lines in (**e**)) in the bZZ-qBI. ZZMI is the abbreviation for the zigzag monolayer ice. Source data are provided as a Source Data file^78^.

Next, we simulated phase transitions from the liquid water with the lateral pressure increasing from 0 to 20 GPa in a step of 1 GPa⋅ns^−1^. Two previously unreported high-density 2D ice phases, namely, quasi-bilayer ices (qBI), were observed (Supplementary Figs. 10–14). Specifically, the phase transition from the 2D water to the quasi-bilayer ice at 300 K, involving two intermediate phases, is shown in Fig. 3a and Supplementary Movie 9. At 300 K, the 2D water transformed from the liquid to hexatic monolayer ice, then to ZZMI and quasi-bilayer ice in succession, as evidenced by the spatial distribution of oxygen and hydrogen atoms. As shown in the top panel of Fig. 3a, oxygen and hydrogen patterns underwent disordered-to-angular distribution with 6-fold rotational symmetry, indicating the formation of the hexatic state as reported by Kapil et al.^48^ The solid-liquid intermediate state of the hexatic phase is consistent with the well-known KTHNY theory^68^ of 2D melting transition. As the lateral pressure increases to 5 GPa, the computed angular distribution of hydrogen atoms suggests the formation of the ZZMI phase. At *P* = 20 GPa, both oxygen and hydrogen present a more concentrated angular distribution. Water molecules were distributed in two sublayers, as evidenced by the oxygen distribution profile along the *z*-axis (normal to the walls; see Fig. 3b), where one single peak split into two separate ones. The continuous variation of the full width at half maximum of the peak (FWHM as defined in Fig. 3b) well described the sublayer-separation process (red lines in Fig. 3a), where two obvious sublayers were observed at FWHM = 0.7 Å. Further analysis of the location of the hydrogen bonds (Fig. 3c) revealed that the hydrogen bonds mainly existed within the separated planar sublayers (corresponding to the intra-layer H-bonding network) but were rarely observed between the two sublayers. This suggests the lack of H-bonds formed between two sublayers. The lack of an inter-layer H-bonding network distinguishes this new high-area-density ice from known monolayer ices. All these results demonstrate the formation of novel quasi-bilayer ice phases with an equilibrium inter-layer distance of 0.8 Å (Fig. 3b).

In the quasi-bilayer ice, two different water arrangement patterns were observed in our MD simulations. Figure 3d presents the atomic structure of one of the two, namely, the zigzag quasi-bilayer ice (ZZ-qBI). The water molecules in each sublayer form zigzag chains. Following the AB stacking rule, the zigzag chains are alternatively arranged in the upper (red) and lower (blue) sublayers. Such water arrangement can be considered the ZZMI puckered along the direction perpendicular to the zigzag chain. Notably, it entails the quasi-bilayer feature without forming the stable inter-sublayer H-bonding network.

Unlike the water arrangement in the ZZ-qBI, the formation of the zigzag chains with branches contains isolated water molecules, thereby named as the branched-zigzag quasi-bilayer ice (bZZ-qBI, Fig. 3e). As shown in Supplementary Movie 10, its stability is confirmed with MLFF-based MD simulations at 15 GPa. In the bZZ-qBI, each zigzag chain (purple lines) is sandwiched between two lines of isolated water molecules, and these lines are named as the branches (orange lines). Each water molecule in the zigzag chain forms a dangling hydrogen bond with a branch water molecule. Like the ZZ-qBI, the upper and lower sublayers with the branched zigzag chains stack together in the AB pattern (see side view in Fig. 3e). Such a water pattern manifests that half of the number of the water molecules only form one hydrogen bond with neighbouring water molecules. The as-formed hydrogen bonds exhibit a large bond angle and are weaker than those in the zigzag chains (Fig. 3f) in which each water molecule has two H-bonding neighbour water molecules. Water molecules in both the zigzag chains and branches possess fewer hydrogen bonds than those in bulk water/ice and thus do not satisfy the ice rule^20^ (which requires each water molecule being hydrogen-bonded with four nearest neighbours). This result suggests that the bZZ-qBI possesses a unique H-bonding network with the coexistence of strong H-bonds in the zigzag chain and weak ones between the zigzag chains and branches.

**Fig. 4:**
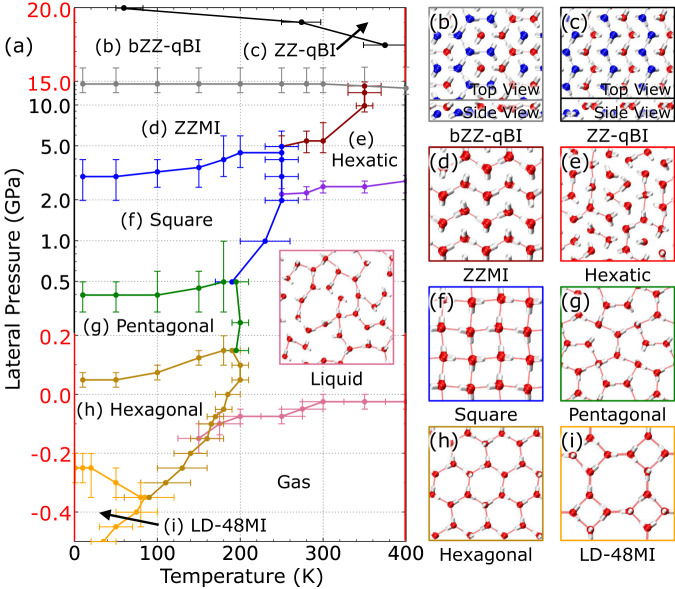
Phase diagram of the 2D water/ice system in nanoconfinement. **a** Temperature-pressure phase diagram of the 2D water in a 6.0 Å wide nanoslit. The vertical axis in black and red are in the logarithm and linear scale, respectively, where the negative pressure region is also included. **b**–**i** Molecular structures of various 2D ices. The same colour code as Fig. 3 is used here. Here, bZZ-qBI, ZZ-qBI, ZZMI, and LD-48MI represent branched zigzag quasi-bilayer ice, zigzag quasi-bilayer ice, zigzag monolayer ice, and low-density 4⋅8^2^ monolayer ice, respectively. The error bar describes uncertainty of the phase boundary, and the specific definition can be found in Section “Determination of phase boundaries”. Source data are provided as a Source Data file^78^.

Overall, we observed two new high-density ices that have a quasi-bilayer structure. Unlike conventional bilayer ices, few inter-layer hydrogen bonds were observed in both quasi-bilayer ices, although both can be also viewed as unconventional monolayer ice. The bZZ-qBI entails a unique H-bonding network that is quite different from that of the ZZ-qBI. We also performed 20-ps AIMD simulations with both vdW-DF2 and rev-PBE0-D3 functionals to examine the stability of both qBIs at 300 K and 15 GPa. As shown in Supplementary Movies 11–14, both ZZ-qBI and bZZ-qBI maintained their original crystal structures during the simulations, confirming their stability. We further performed vdW-DF2-based AIMD simulations with realistic graphene walls at 300 K (Supplementary Movies 15, 16) and 350 K (Supplementary Movies 17, 18). The simulations show that both 2D ices are stable in the graphene nanoslit. In addition, we conducted AIMD simulations with realistic graphene walls using BLYP-D3 (Supplementary Movies 19, 20) and rev-PBE0-D3 (Supplementary Movie 21) functionals to check functional dependency and confirmed their stability. To examine the nuclear quantum effect, we also performed path-integral^69^ MD (PIMD) simulations using the MLFF model. The PIMD simulations indicate that both 2D ices are still stable with little structure changes^69,70^ (see Supplementary Movies 22, 23). Furthermore, the independent free-energy calculations show that the bZZ-qBI is more stable than the ZZ-qBI at *P* < 19 GPa (Supplementary Fig. 16). Nonetheless, we only observed the partial formation of the bZZ-qBI (Supplementary Fig. 17a), probably due to the memory effect of the system during the phase transition from the ZZMI (being an adjacent phase below 15 GPa in the phase diagram as shown in Fig. 4) to the qBI.

The nanoslit width can affect the phase behaviour of the 2D water. To demonstrate the width effect, we performed an independent series of MLFF-based MD simulations with nanoslits of 5.0, 5.5 and 6.5 Å width, respectively. The simulations show that both ZZ-qBI and bZZ-qBI are also stable within a nanoslit of 6.5 Å width at pressure of 8–10 GPa and 10–15 GPa, respectively (see Supplementary Movies 24, 25). Within a nanoslit of 5.5 Å width, much higher pressure (>50 GPa) is required to maintain the stability of ZZ-qBI and bZZ-qBI (see Supplementary Movies 26, 27). Within a nanoslit with 5.0 Å width, the initial qBI quickly transforms into the ZZMI phase based on PIMD simulations (see Supplementary Movie 28).

MD simulations have shown the existence of various monolayer ice and qBI phases of the 2D water. Both first-order and continuous phase transitions were observed. Specifically, a sudden change of the potential energies was observed in the phase transitions of ZZMI-to-hexatic, square-to-liquid, pentagonal-to-liquid and hexagonal-to-liquid, suggesting strong first-order phase transitions (Supplementary Fig. 18a–d). Unequal derivatives of the free energy of two phases at the transition point also indicated the first-order transition for the pentagonal-to-hexagonal (Supplementary Fig. 19a), pentagonal-to-square (Supplementary Fig. 19b) and bZZ-qBI-to-ZZ-qBI (Supplementary Fig. 16) transitions. In contrast, the ZZMI-to-bZZ-qBI transition was likely a continuous phase transition due to continuous variation of potential energy with lateral pressure (Supplementary Fig. 18e). A similar phase behaviour was seen for the liquid-to-hexatic transition (Supplementary Fig. 18f), consistent with Kapil et al.’s study^48^.

### Phase diagram of 2D monolayer ices

Next, we computed the *T-P* phase diagram of the monolayer water in the range of 0–400 K and −0.5 to 20 GPa to confirm the stable region of each phase (see the section ‘Determination of phase boundaries’ for more details). Given the real-time observation of the freezing transitions in the MD simulations, the reversible phase transitions by changing either the temperature or the lateral pressure reversibly were also conducted in the MD simulations to estimate the solid-liquid phase boundary. In most cases, the reversible solid-to-solid transitions are difficult to achieve in MD simulations. Hence, the thermodynamic integration method with the Einstein crystal as the reference was also used to compute the free energy changes of the solid-to-solid transitions so that the corresponding phase boundary can be determined. The liquid-gas and solid-gas boundaries were identified when the H-bonding network in the liquid and solid was about to collapse. The computed phase diagram and the accompanying crystal structure of each phase are shown in Fig. 4. Note that we also employed the random structure search method^71^ to search new 2D low-density ice structures. Two potential 2D ice structures, namely, 4 ⋅ 6 ⋅ 8 and 5 ⋅ 8 (Supplementary Fig. 20a, c), were identified. Further MLFF-based MD simulations indicate that the former is unstable in the phase region of LD-48MI, while the latter is a metastable phase (Supplementary Fig. 20b, d).

As shown in Fig. 4, the liquid phase is adjacent to four solid phases, i.e., hexagonal, pentagonal, square and hexatic monolayer ices, similar to the phase diagram reported by Kapil et al.^48^ A phase boundary between the square and hexatic monolayer ices was obtained in the phase diagram. Four triple points of the 2D water were identified at (–0.1 GPa, 160 K), (0.12 GPa, 190 K), (0.5 GPa, 190 K) and (2.0 GPa, 240 K), respectively, where the 2D water reaches equilibrium with the other two adjacent phases. The triple point at (–0.1 GPa, 160 K) suggests that the gas, monolayer liquid and solid can coexist in a nano-confined space like that in the 3D space. At temperatures below 200 K, the hexagonal, pentagonal and square monolayer ice phases become the most stable in succession. This result confirms the real-time observation of our MD simulations involving solid-to-solid transitions (Fig. 2c and Supplementary Fig. 7). At *P* > 3 GPa, the square monolayer ice is expected to transform to the ZZMI, consistent with the real-time MD simulations (Supplementary Fig. 8). An increase in temperature leads to the transition of ZZMI to the hexatic monolayer ice phase. The square, zigzag and hexatic monolayer ice phases can reach equilibrium at 5.0 GPa and 240 K. Further increase of the pressure to 15 GPa leads to the formation of the bZZ-qBI, whereas the ZZ-qBI becomes the most stable for *P* beyond 18 GPa but can also be a meta-stable phase in the bZZ-qBI domain region (Supplementary Fig. 16). Besides, we performed AIMD simulations to verify the stability of the hexagonal (Supplementary Movie 29), pentagonal (Supplementary Movie 30), square (Supplementary Movie 31) and ZZMI (Supplementary Movie 32), in the corresponding phase region. For these simulations, the *NPT* ensemble was used.

More importantly, we identified a stable region for the low-area-density monolayer ice, the LD-48MI. The LD-48MI was first reported in 2010^21^, but its stability relative to other phases has been unclear. The phase diagram shown in Fig. 4 clearly shows that the LD-48MI phase becomes the most stable at the negative pressure region of *P* < − 0.3 GPa for *T* < 90 K. We further confirmed its stability at (−0.4 GPa, 50 K) via independent AIMD simulations with 64 water molecules confined in realistic graphene nanoslits (Supplementary Movie 33). In the gas-phase region, the LD-48MI (Supplementary Movies 34, 35) and hexagonal ice (Supplementary Movie 36) collapsed, as observed in the AIMD simulations. The stable region reported here provides a guide for controlling the negative pressure in the future experimental realization of the LD-48MI.

We have performed a comprehensive large-scale simulation study of the phase behaviour of 2D water/ices based on the developed MLFF model. The MLFF model was proven at the first-principles accuracy and reproduced the DFT *E*-*A* curves of the 2D ices. Through the MLFF-based MD simulations, we observed, in real-time, the spontaneous formation of 2D ices from liquid 2D water and confirmed the stability of the ZZMI, square, pentagonal and hexagonal monolayer ices. More importantly, we observed two previously unreported high-density ices, ZZ-qBI and bZZ-qBI, both exhibiting the quasi-bilayer structure with few inter-layer H-bonds. The ZZ-qBI can be viewed as the ZZMI puckered along the direction perpendicular to the zigzag direction. Notably, a unique H-bonding network with weak dangling hydrogen bonds was characterized for the bZZ-qBI.

We have also constructed a full phase diagram of 2D water/ices in the range of –0.5 GPa ≤ *P* ≤ 20 GPa and for *T* ≤ 400 K by combining the MD simulations with the thermodynamic integration method. In particular, the phase diagram comprises the low-density $4\cdot {8}^{2}$ monolayer ice in the negative pressure region and the high-density quasi-bilayer ices in the high-pressure region. The free-energy computation shows quantitatively that the LD-48MI becomes the most stable at pressure <−0.3 GPa. Overall, our comprehensive studies not only have verified the stabilities of known monolayer ices but also predicted two new high-pressure qBI phases, as well as identified negative pressure region for the low-density monolayer ice phase. These MLFF-based MD simulations of monolayer phase transitions provide a generic framework for future study of the phase behaviour of multilayer water/ices.

## Methods

### Reference DFT calculations

An active-learning approach shown in Fig. 1a was used for reference data collection (see the supplementary information for more details). The reference data were generated using the Gaussian and plane-wave methods implemented in the CP2K Quickstep package^72^. The Goedecker-Teter-Hutter (GTH) pseudopotentials and TZV2P-MOLOPT-GTH basis sets were employed to simulate the core and valence electrons for the O and H atoms, respectively. The energy cutoffs for the finest grid level and Gaussian waves were 800 and 50 Ry, respectively. The vdW-DF2 exchange-correlation functional (also named the PW86-vdW2 functional^27^) was adopted to describe the exchange and correlation interactions. This functional has been proven to show the best result (compared to the quantum Monte Carlo simulation) in simulating the 2D ices by Brandenburg et al.^66^ Note that Brandenburg et al. evaluated performance of DFT functionals for various systems, including 2D/3D ices, water molecules on graphene, in carbon nanotubes, and on aromatic hydrocarbon sheets. Our research focuses on the phase behaviour of the 2D ices. Thus, the vdW2-DF2 functional was selected as the reference, on the basis of error analysis shown in the 4th and 5th columns of Table 4 in ref. ^66^.

### Development and validation of the MLFF model

The DeePMD-kit package^42^ was used to train the MLFF model. A neural network of 25 × 50 × 100 was adopted in the descriptor section and a single hidden layer with 240 neurons in the fitting section. A cutoff distance of 9.0 Å with a smoothing value of 1.0 Å was employed for training. All other parameters were set as those provided in the water example of the DeePMD-kit^42,73^. In total, 25,623 atomic images were included for the development of the productive MLFF model. The atomic images of the liquid phase of 2D water generated from the finite-temperature MD simulations were also included (Supplementary Fig. 21). The ratio of the training and test data was set as 3:1 to avoid overfitting.

We evaluated the derived MLFF model with validation data sampled from AIMD simulations of solid, liquid and gas phases of 2D ices and from MLFF-based MD simulations of freezing, melting and solid-solid transition processes discussed above. Note that the AIMD simulations used for validation data collection were performed to validate the phase stability of the 2D ice/water system in their respective stable region (see Supplementary Movies 29–33), independent from those used in the active-learning processes. Atomic images of the two predicted quasi-bilayer ices, ZZ-qBI and bZZ-qBI, are also considered. A total number of 11,217 atomic images were collected for the validation (see Supplementary Fig. 22). The obtained root-mean-square errors (RMSE) of energies and forces were 12.2 meV⋅H_2_O^−1^ and 96.7 meV⋅Å^−1^, respectively (see Supplementary Fig. 23a, b), comparable with those of the training data and thus confirming the reliability of our MLFF model. We also computed the *E-A* curves of the ZZ-qBI and bZZ-qBI with the vdW-DF2 functional and our MLFF model. The obtained *E-A* curve from the MLFF model shows good agreement with the one from the vdW-DF2 functional (Supplementary Fig. 24).

We further applied Behler-Parrinello^74^ (BP) symmetry functions (SFs) implemented in n2p2^43^ program to analyze the distribution of local environments of the training data, as well as those of ZZ-qBI and bZZ-qBI. The symmetry function parameters used in Ref. ^48^. were employed here. The results (Supplementary Figs. 25, 26) indicated that the training data (blue bar) cover most of the local environments of the two quasi-bilayer phases (red bar). Representative structures of the qBI in the training data are shown in Supplementary Fig. 27. The outcome explains why our MLFF model gives reasonable results for prediction of the new 2D ice phases. Note that in our MLFF model, the local environments involved in the superionic phase (a stable phase above 400 K) were not included in the training data. Hence, we did not consider temperatures higher than 400 K to analyze the superionic state. As all simulations were performed with temperature below 400 K, no O-H dissociation event was observed.

We also utilized the newly developed MLFF model to simulate the melting process of bulk ice-Ih. Our results, as illustrated in Supplementary Fig. 2, indicate that the ice-Ih melting process started at 230 K, about 40 K lower than the experimental value. This magnitude of underestimation is comparable to that reported in the previous study^73^, which overestimated the melting point by about 40 K. This underestimation by our MLFF model is mainly attributed to the lack of 3D water/ice configurations during the data training and the use of the MLFF model beyond the range of the training data. Thus, we note that the current MLFF model is only applicable to the 2D water/ice system, and it should be used with caution for other water/ice systems, including bulk ice Ih.

### Illustration of the active-learning approach

The present MLFF model was developed with training data collected from an active-learning approach as shown in Fig. 1a. Here, we present a step-by-step illustration of the active-learning approach.

Step 1. An initial MLFF model was trained based on atomic images collected from MD simulations of freezing processes of 2D liquid water reported in a previous study^20^, including AIMD simulations of 2D water confined in nanoslits with width in the range of 6.0 Å to 8.0 Å.

Step 2. The accuracy of the MLFF model was evaluated by comparing the energy-area (*E*-*A*) curves of various 2D ices, including LD-48MI, hexagonal, pentagonal, square monolayer ices and ZZMI, obtained from the MLFF model and the reference DFT method.

Step 3. Representative images were selected at the place where the MLFF *E-A* curve deviated from the DFT value if the expected accuracy of the MLFF model was not reached.

Step 4. AIMD and MLFF-based MD simulations with the selected images as initial configurations at constant volume with temperatures ranging from 10 − 400 K were performed. In addition, MLFF-based MD simulations of the freezing of 2D liquid water and melting of 2D ices were conducted.

Step 5. Images from Step 4 were sampled to balance the distribution of training data on the *E*-*A* curve. The energy and forces of the selected images were evaluated with the reference DFT method for those generated by the MLFF-based MD simulations.

Step 6. A new MLFF model was developed based on the new training data.

Step 7. Repeat Steps 2–6 until the expected accuracy of the MLFF model was reached.

### Implements of LJ93 virtual walls and calculation of lateral pressures

In all simulations reported here (except wherever specified), the interaction between the walls of the nanoslit and O atoms of water molecules was modelled by the 9-3 Lennard-Jones (LJ93) potential described by the following equation^20,22,28,31^,

2 $${E}_{{{{{{\rm{OW}}}}}}}({r}_{{{{{{\rm{OW}}}}}}})=4{\varepsilon }_{{{{{{\rm{OW}}}}}}}\left[{\left(\frac{{\sigma }_{{{{{{\rm{OW}}}}}}}}{{r}_{{{{{{\rm{OW}}}}}}}}\right)}^{9}-{\left(\frac{{\sigma }_{{{{{{\rm{OW}}}}}}}}{{r}_{{{{{{\rm{OW}}}}}}}}\right)}^{3}\right]$$

where *r*_OW_ is the distance between O atoms and the walls. The wall parameters *ε*_OW_ and *σ*_OW_ were set as 2.569 kcal⋅mol^−1^ and 2.754 Å, respectively, which were fitted to reproduce the water-graphene interaction^20^. The open-source PLUMED code^75^ was used to add the LJ93 walls in AIMD simulations. Supplementary Fig. 28 shows a comparison of a nanoslit formed by LJ93 smooth walls with that by realistic graphene walls. The LJ93 nanoslit can mimic the interaction between water molecules and the graphene nanoslit quite well.

In MD simulations with LAMMPS^76^ and CP2K^72^ software packages, the stress tensor elements (${\sigma }_{{{{{{\rm{xx}}}}}}}^{{\prime} }$ and ${\sigma }_{{{{{{\rm{yy}}}}}}}^{{\prime} }$) of a slab-vacuum model were calculated over the whole simulation box, including the vacuum. Hence, they are not equivalent to the lateral pressure applied to the slab (nanoslit-encapsulated 2D water). As shown in previous study^19^, the effective lateral pressure applied to the slab can be calculated as $P=({\sigma }_{{{{{{\rm{xx}}}}}}}+{\sigma }_{{{{{{\rm{yy}}}}}}})/2$, where *σ*_xx_ and *σ*_yy_ are calculated with ${{{{{\rm{\sigma }}}}}}=\sigma ^{\prime} \times {L}_{z}/{{{{{\rm{w}}}}}}$. Here, *L*_*z*_ is the *z*-axis length of the simulation box, and *w* is the width of the nanoslit, i.e., 6.0 Å in this work.

### Determination of phase boundaries

Solid-to-solid phase boundaries were determined by calculating the Gibbs free energies of the two phases using thermodynamic integration methods^77^. At a given temperature and pressure, the Helmholtz free energy ${F}_{{{{{{\rm{2D}}}}}}{{{{{\rm{ice}}}}}}}$ of a solid phase was computed with the following equation,

3 $${F}_{{{{{{\rm{2D}}}}}}\, {{{{{\rm{ice}}}}}}}={F}_{{{{{{\rm{Einstein}}}}}}}\,+\overline{{W}_{{{{{{\rm{Einstein}}}}}}}^{{{{{{\rm{2D}}}}}}\,{{{{{\rm{ice}}}}}}}}$$

where ${F}_{{{{{{\rm{Einstein}}}}}}}$ is the Helmholtz free energy of the corresponding Einstein crystal. The spring constant in the Einstein crystal was calculated with $k=100\frac{3{k}_{B}T}{\langle {(\varDelta r)}^{2}\rangle }$, where *k*_*B*_, *T* and $\langle {(\varDelta r)}^{2}\rangle$ are the Boltzmann constant, temperature, and mean-squared displacement of all atoms in the system. The integration $\overline{{W}_{{{{{{\rm{Einstein}}}}}}}^{{{{{{\rm{2D}}}}}}\,{{{{{\rm{ice}}}}}}}}={\int }_{0}^{1}d\lambda \langle {\frac{\partial H(\lambda )}{\partial \lambda }}\rangle_{\lambda }$ is defined along a reversible scaling path by a parametrical Hamiltonian $H(\lambda )=\lambda {H}_{{{{{{\rm{2D}}}}}}\, {{{{{\rm{ice}}}}}}}+(1-\lambda ){H}_{{{{{{\rm{Einstein}}}}}}}$. *λ* is a scaling factor in the potential energy function and computed with the following switching function:

4 $$\lambda (\tau )={\tau }^{5}(70{\tau }^{4}-315{\tau }^{3}+540{\tau }^{2}-420\tau+126)$$

where $\tau=t/{t}_{s}$ and *t* represents the current step number. *t*_*s*_ is the number of steps required for the switching procedure and set to 200,000. 50,000 MD steps were performed at the equilibration stage to guarantee the convergence of the simulation. This method is applicable for ordered crystalline systems^77^. As reported in ref. ^77^, we computed the Helmholtz free energies for both the forward ($\lambda :0\to 1$) and backward ($\lambda :1\to 0$) processes. Next the corresponding Gibbs free energy for each process was calculated as $G={F}_{{{{{{\rm{2D}}}}}}\, {{{{{\rm{ice}}}}}}}+{{{{{\rm{PV}}}}}}$, where *V* is the volume of the nanoslit. The Gibbs free energy of the phase transition was averaged over the forward and backward processes, and the error is determined as the difference in Gibbs free energy between the two processes^77^. Note that the ZZMI-qBI phase boundary (the grey line in Fig. 4) was calculated with the following slow pressure ramp method. The key structure parameter, FWHM, used for distinguishing ZZMI and qBI entails a continuous variation with pressure (Fig. 3a) during the transition.

The gas-related, liquid-related, hexatic-related and ZZMI-qBI phase boundaries were computed with slow temperature/pressure ramp methods. Take the melting process as an example, we first stabilized the initial configuration at a given temperature and pressure for at least 1 ns. Then the temperature was increased at a rate of 100 K⋅ns^−1^ until observation of a melting process. The temperature was then fixed at the melting point for at least 1 ns to stabilize the system. A similar approach was applied to determine the gas-related boundary via pressure variation at a rate of 1 GPa⋅ns^−1^ to simulate the sublimation or evaporation processes.

Due to the statistical and systematic errors, the comparison of free energies of two phases was non-deterministic in a certain region. This region was viewed as an uncertainty region. The error bar was considered to be the temperature/pressure range of the uncertainty region. In the temperature/pressure ramp method, we scanned over the temperature/pressure region of the phase diagram and performed the MD simulation at each scan point. The phase transition event was observed in a certain temperature/pressure interval which was considered as the uncertainty region. With using the thermodynamic integration method, free energies of two phases were used to determine the phase boundary. Using the phase boundary of the bZZ-qBI and ZZ-qBI as an example (Supplementary Fig. 16b), we conducted calculations to construct the temperature-dependent free-energy curve for both phases at the given pressure of 17.5 GPa. The phase boundary was identified at the cross point of the two curves, i.e., ~375 K. Note that the free energy at the cross point was estimated through linear interpolation, and therefore an error bar was incorporated. The lower and upper limits of the error bar were determined by considering the nearest points, with values of 350 K and 400 K, respectively. The same method was employed to calculate the error bar associated with the pressure.

### Additional computational details

The MD simulations based on the MLFF model and ab initio methods were performed using the LAMMPS^76^ and CP2K^72^ package, respectively. The related input data and scripts were provided^78^. A timestep of 1 fs was used in both simulations. A 2D ice/water model with 192 water molecules was employed in all MLFF-based MD simulations. Additional PIMD simulations were carried out by using the i-PI^69^ code to examine the nuclear quantum effects (see Supplementary information for more details). The atomic simulation environment (ASE)^79^ package was used to read atomic structures from LAMMPS and CP2K outputs for data analysis. The visual molecular dynamics (VMD)^80^ package and OVITO^81^ tool were used to visualize atomic structures. The mean square displacement (MSD) at time *t* was calculated according to Eq. (5):

5 $${{{{{\rm{MSD}}}}}}(t)=\frac{1}{{{{{{\rm{natoms}}}}}}}\mathop{\sum }\limits_{i=0}^{{{{{{\rm{natoms}}}}}}}{({r}_{i}^{t}\mbox{-}{r}_{i}^{0})}^{2}$$

where *r*_*i*_^*t*^ and *r*_*i*_^0^ are the positions of atom *i* at time *t* and 0, respectively. The histograms of <*z* > -coordinates for O atoms with a bin of 0.1 Å were calculated by NumPy^82^. Then the Gaussian kernel density evaluation (KDE) of SciPy^83^ was adopted to fit the histograms to obtain smooth distribution functions. All figures and movies were created with the Matplotlib^84^ package.

PIMD simulations based on the MLFF model including 8 beads were performed to evaluate the nuclear quantum effects, using the LAMMPS package. The *NVT* ensemble with a timestep of 0.25 fs was adopted. The system volume was set as the equilibrium volume from the corresponding *NPT* AIMD simulations. The LJ93 smooth walls were adopted for the nanoslits. A 12-6 Lennard-Jones force with the following equation was applied when the O-H distance (*r*_OH_) was less than 0.8 Å to avoid the collapse of the MLFF model beyond the range of the training data:

6 $${E}_{{{{{{\rm{OH}}}}}}}({r}_{{{{{{\rm{OH}}}}}}})=4{\varepsilon }_{{{{{{\rm{OH}}}}}}}\left[{\left(\frac{{\sigma }_{{{{{{\rm{OH}}}}}}}}{{r}_{{{{{{\rm{OH}}}}}}}}\right)}^{12}-{\left(\frac{{\sigma }_{{{{{{\rm{OH}}}}}}}}{{r}_{{{{{{\rm{OH}}}}}}}}\right)}^{6}\right]$$

Here, the wall parameters *ε*_OH_ and *σ*_OH_ were set as 0.231 kcal⋅mol^−1^ and 0.8 Å, respectively.

### Reporting summary

Further information on research design is available in the Nature Portfolio Reporting Summary linked to this article.

## Supplementary information

Supplementary Information

Description of Additional Supplementary Files

Supplementary Movie 1

Supplementary Movie 2

Supplementary Movie 3

Supplementary Movie 4

Supplementary Movie 5

Supplementary Movie 6

Supplementary Movie 7

Supplementary Movie 8

Supplementary Movie 9

Supplementary Movie 10

Supplementary Movie 11

Supplementary Movie 12

Supplementary Movie 13

Supplementary Movie 14

Supplementary Movie 15

Supplementary Movie 16

Supplementary Movie 17

Supplementary Movie 18

Supplementary Movie 19

Supplementary Movie 20

Supplementary Movie 21

Supplementary Movie 22

Supplementary Movie 23

Supplementary Movie 24

Supplementary Movie 25

Supplementary Movie 26

Supplementary Movie 27

Supplementary Movie 28

Supplementary Movie 29

Supplementary Movie 30

Supplementary Movie 31

Supplementary Movie 32

Supplementary Movie 33

Supplementary Movie 34

Supplementary Movie 35

Supplementary Movie 36

## Data Availability

The data, such as atomic structures, the machine-learning force field and so on, required to reproduce the key findings of this work are available at GitHub repository (https://github.com/leilist/Monolayer-Water-PhaseDiagram-Data) and Zenodo^78^. More detailed data are available from the corresponding author upon request. Source data^85^ are provided with this paper. Source data are provided with this paper.

## Source data

Source Data

## Code availability

The input scripts and codes to reproduce the key findings of this work are available at GitHub repository (https://github.com/leilist/Monolayer-Water-PhaseDiagram-Data) and Zenodo^78^. More detailed data are available from the corresponding author upon request.
