# Supplementary material for: Temperature-pressure phase diagram of confined monolayer water/ice at first-principles accuracy with a machine-learning force field
Source: Nat Commun. 2023 Jul 11;14:4110. doi: 10.1038/s41467-023-39829-z (PMC10336112; doi:10.1038/s41467-023-39829-z)
Supplement: Supplementary file 1 — Supplementary Information [file 41467_2023_39829_MOESM1_ESM.docx]

**Supplementary Information**

**Temperature-Pressure Phase Diagram of Confined Monolayer Water/Ice at First-Principles Accuracy with a Machine-Learning Force Field**

Bo Lin ^1^, Jian Jiang ^2,3^, Xiao Cheng Zeng ^2,3^*, Lei Li ^1,^*

^1^Shenzhen Key Laboratory of Micro/Nano-Porous Functional Materials (SKLPM), Department of Materials Science and Engineering, Southern University of Science and Technology, Shenzhen, 518055, China; ^2^Department of Materials Science and Engineering, City University of Hong Kong, Kowloon, 999077, Hong Kong; ^3^Department of Chemistry, University of Nebraska-Lincoln, Lincoln, Nebraska, 68588, USA

B.L. and J.J. contributed equally to this work

^*^To whom correspondence may be addressed.

Xiao Cheng Zeng, and Lei Li

**Email:** xzeng26@cityu.edu.hk or [lil33@sustech.edu.cn](mailto:lil33@sustech.edu.cn)


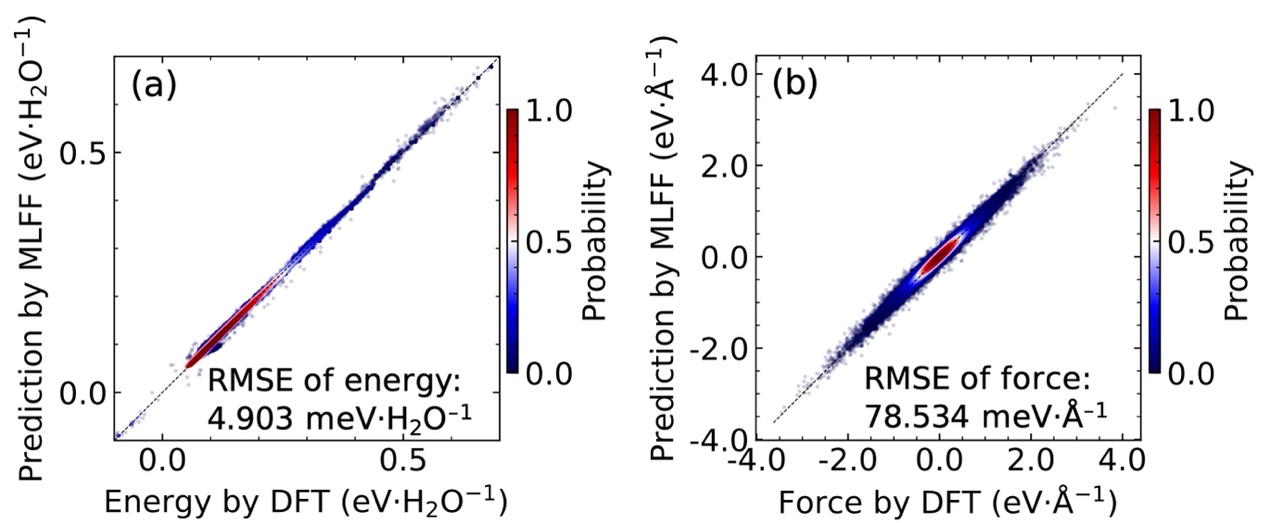


**Supplementary Fig. 1** Performance of the machine-learning force field (MLFF) model. Prediction-true plots on (a) energy and (b) atomic forces. The color represents the probability distribution of the “true-prediction” points. The probability decreases from red to blue. DFT and RMSE are the abbreviations for density functional theory and root-mean-square error, respectively.

**
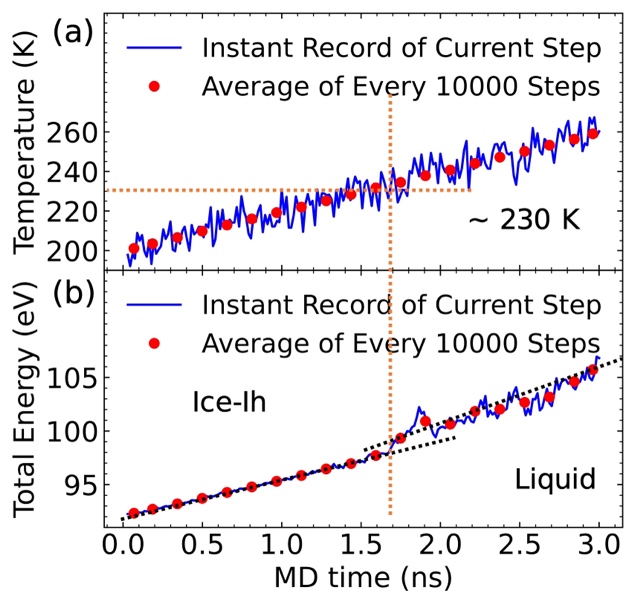
**

**Supplementary Fig. 2** The melting process of bulk ice-Ih obtained with the newly trained machine-learning force field (MLFF) model. (a) The temperature evolution and (b) the total energy evolution by molecular dynamics (MD) simulations.


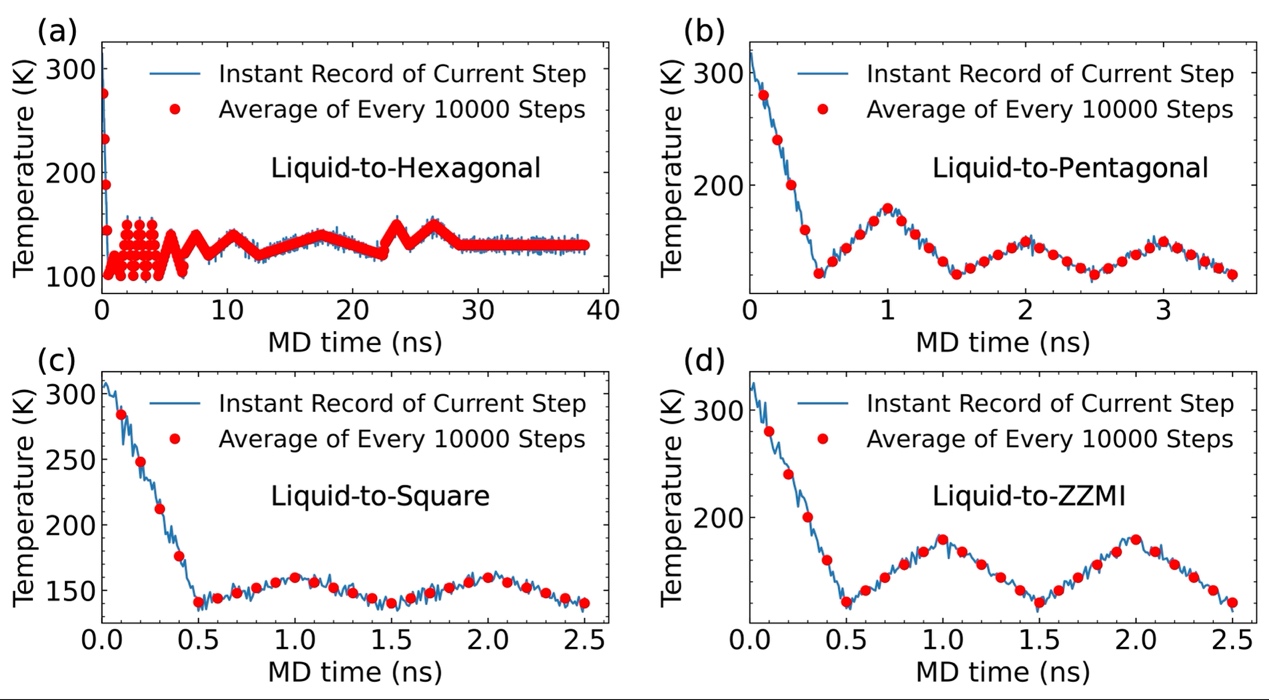


**Supplementary Fig. 3** Annealing processes from molecular dynamics (MD) simulations. Temperature evolution in the annealing process during the transition of the 2D liquid to the (a) hexagonal, (b) pentagonal, (c) square monolayer ice, and (d) ZZMI, respectively.


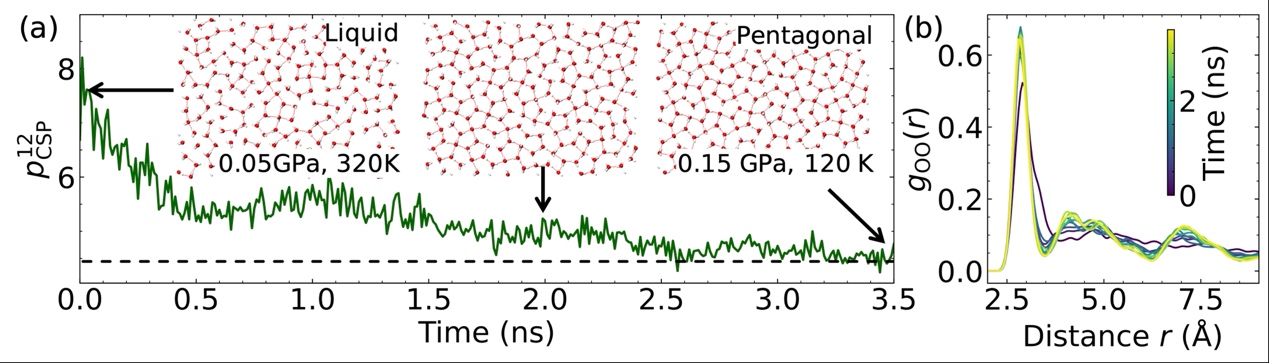


**Supplementary Fig. 4** Liquid-to-pentagonal transition. (a) The evolution of the centro-symmetry parameter (CSP) of the water molecule with 12 nearest oxygen atoms, $\text{p}_{\text{CSP}}^{\text{12}}$, and (b) the pair distribution function of the oxygen atoms, $\text{g}_{\text{OO}}\text{(}\text{r}\text{)}$, during the molecular dynamics (MD) simulation of spontaneous formation of pentagonal ice from the 2D water. The same color code as in Fig. 2 is used. $\text{p}_{\text{CSP}}^{\text{12}}$ is defined in Eq.1 with $N=12$.


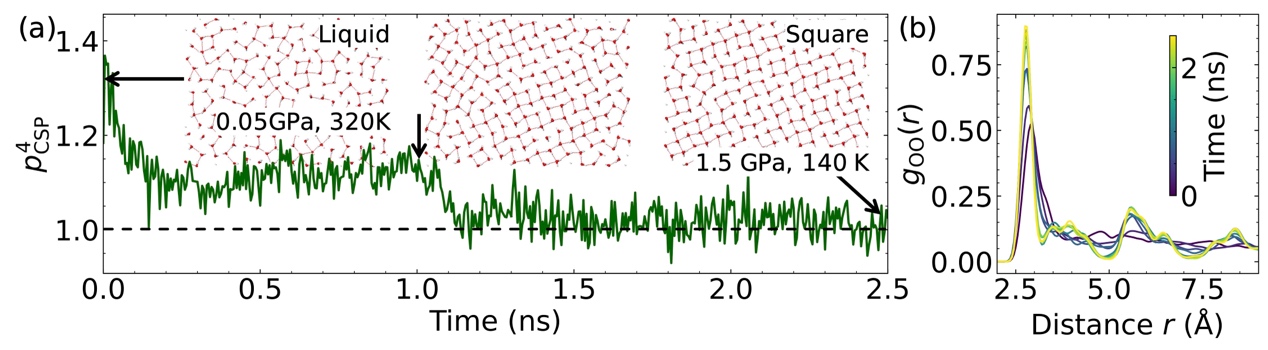


**Supplementary Fig. 5** Liquid-to-square transition. (a) The evolution of the centro-symmetry parameter (CSP) of the water molecule with four nearest oxygen atoms, $\text{p}_{\text{CSP}}^{\text{4}}$, and (b) the pair distribution function of the oxygen atoms, $\text{g}_{\text{OO}}\text{(}\text{r}\text{)}$, during the molecular dynamics (MD) simulation of spontaneous formation of square ice from the 2D water. The same color code as in Fig. 2 is used. $\text{p}_{\text{CSP}}^{\text{4}}$ is defined in Eq.1 with $N=4$.


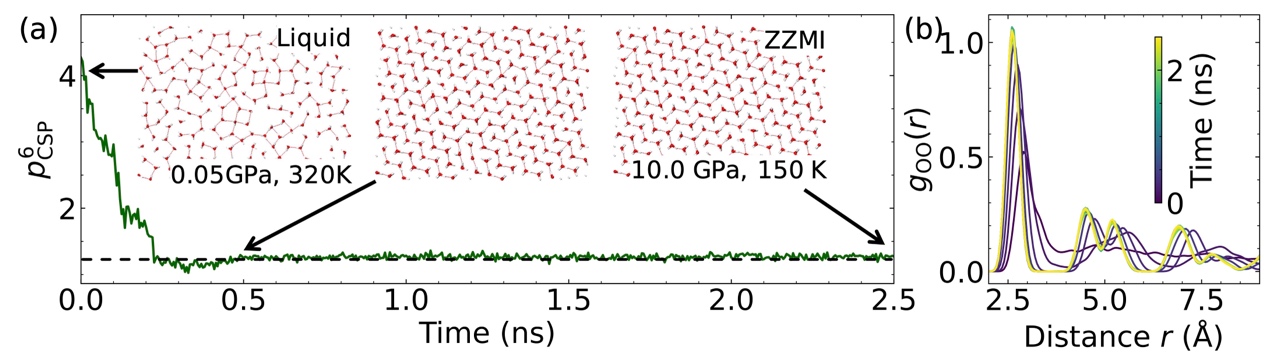


**Supplementary Fig. 6** Liquid-to-ZZMI (zigzag monolayer ice) transition. (a) The evolution of the centro-symmetry parameter (CSP) of the water molecule with six nearest oxygen atoms, $\text{p}_{\text{CSP}}^{\text{6}}$, and (b) the pair distribution function of the oxygen atoms, $\text{g}_{\text{OO}}\text{(}\text{r}\text{)}$, during the molecular dynamics (MD) simulation of spontaneous formation of ZZMI ice from the 2D water. The same color code as in Fig. 2 is used. $\text{p}_{\text{CSP}}^{\text{6}}$ is defined in Eq.1 $N=6$.

**
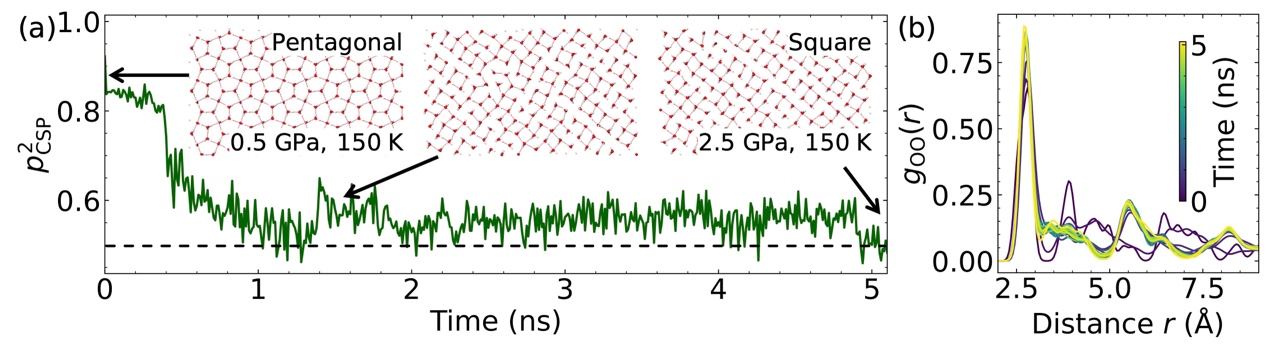
**

**Supplementary Fig. 7** Pentagonal-to-square transition. (a) The evolution of the centro-symmetry parameter of the water molecule with two nearest oxygen atoms, $\text{p}_{\text{CSP}}^{\text{2}}$, and (b) the pair distribution function of the oxygen atoms, $\text{g}_{\text{OO}}\text{(}\text{r}\text{)}$, during the molecular dynamics (MD) simulation of the pentagonal-to-square transition. The same color code as in Fig. 2 is used. $\text{p}_{\text{CSP}}^{\text{2}}$ is defined in Eq.1 $N=2$.


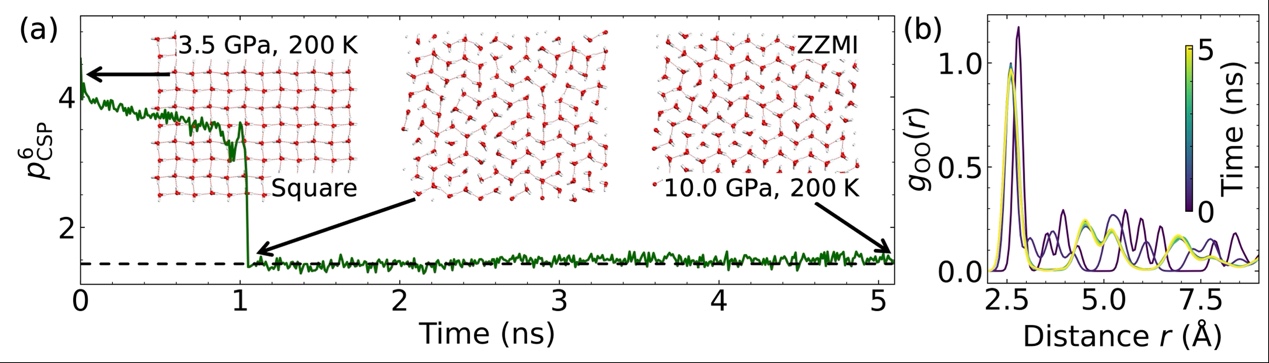


**Supplementary Fig. 8** Square-to-ZZMI (zigzag monolayer ice) transition. (a) The evolution of the centro-symmetry parameter of the water molecule with six nearest oxygen atoms, $\text{p}_{\text{CSP}}^{\text{6}}$, and (b) the pair distribution function of the oxygen atoms, $\text{g}_{\text{OO}}\text{(}\text{r}\text{)}$, during the molecular dynamics (MD) simulation of the square-to-ZZMI transition. The same color code as in Fig. 2 is used. $\text{p}_{\text{CSP}}^{\text{6}}$ is defined in Eq.1 with $N=6$.


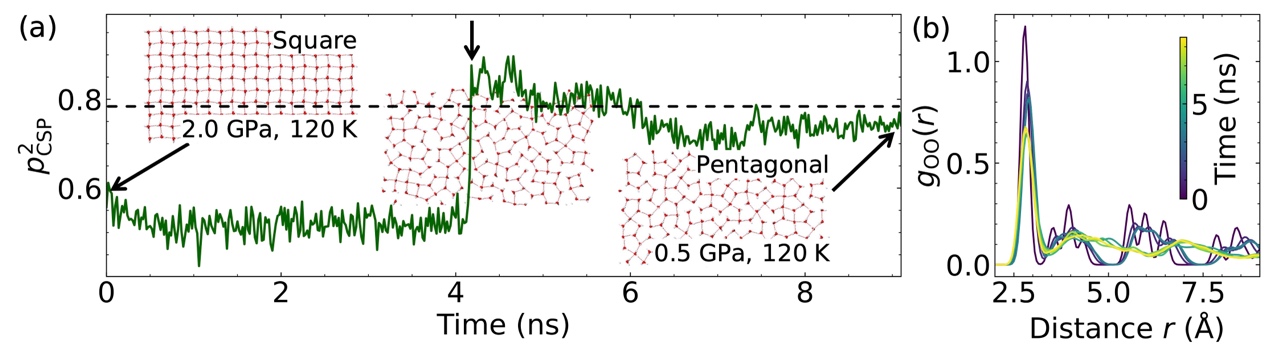


**Supplementary Fig. 9** Square-to-pentagonal transition. (a) The evolution of the centro-symmetry parameter of the water molecule with two nearest oxygen atoms, $\text{p}_{\text{CSP}}^{\text{2}}$, and (b) the pair distribution function of the oxygen atoms, $\text{g}_{\text{OO}}\text{(}\text{r}\text{)}$, during the molecular dynamics (MD) simulation of the square-to-pentagonal transition. The same color code as in Fig. 2 is used. $\text{p}_{\text{CSP}}^{\text{2}}$ is defined in Eq.1 $N=2$.


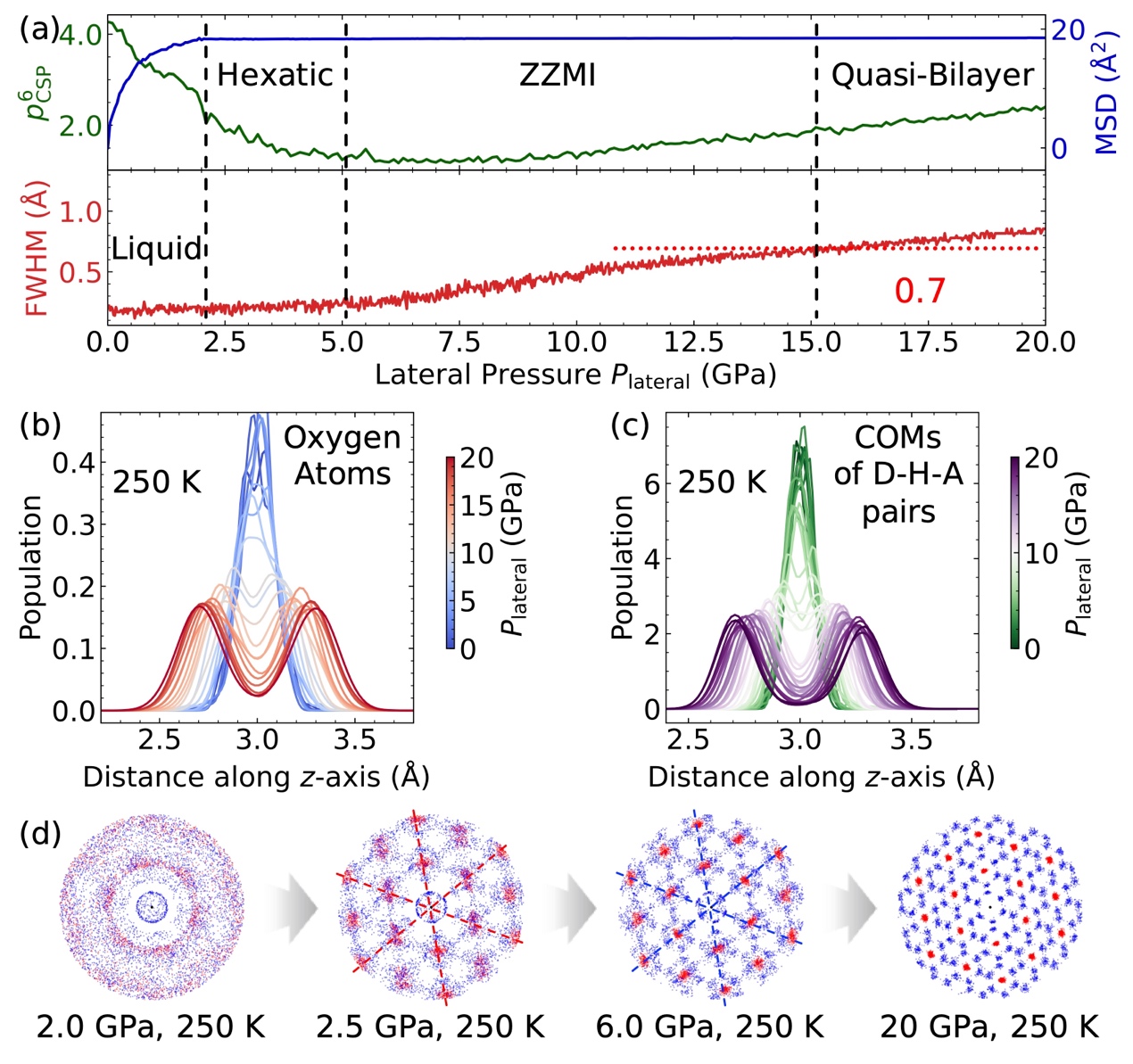


**Supplementary Fig. 10** Molecular dynamics (MD) simulations of sequential phase transition of the 2D water at 250 K. (a) Variation of centro-symmetry parameter (CSP) with six nearest oxygen atoms ($\text{p}_{\text{CSP}}^{\text{6}}$, green curves), mean squared displacement (MSD, blue curves) and full width at half maximum (FWHM, red curves) of the distribution function defined in (b). (c) The distribution functions of center-of-mass (COM) of donor-hydrogen-acceptor (D-H-A) pairs along the *z*-axis evolve with the lateral pressure. (d) Spatial distributions of oxygen (red dots) and hydrogen atoms (blue dots) within a cutoff of 6 Å at different time slots of the MD simulation. ZZMI is the abbreviation for zigzag monolayer ice.


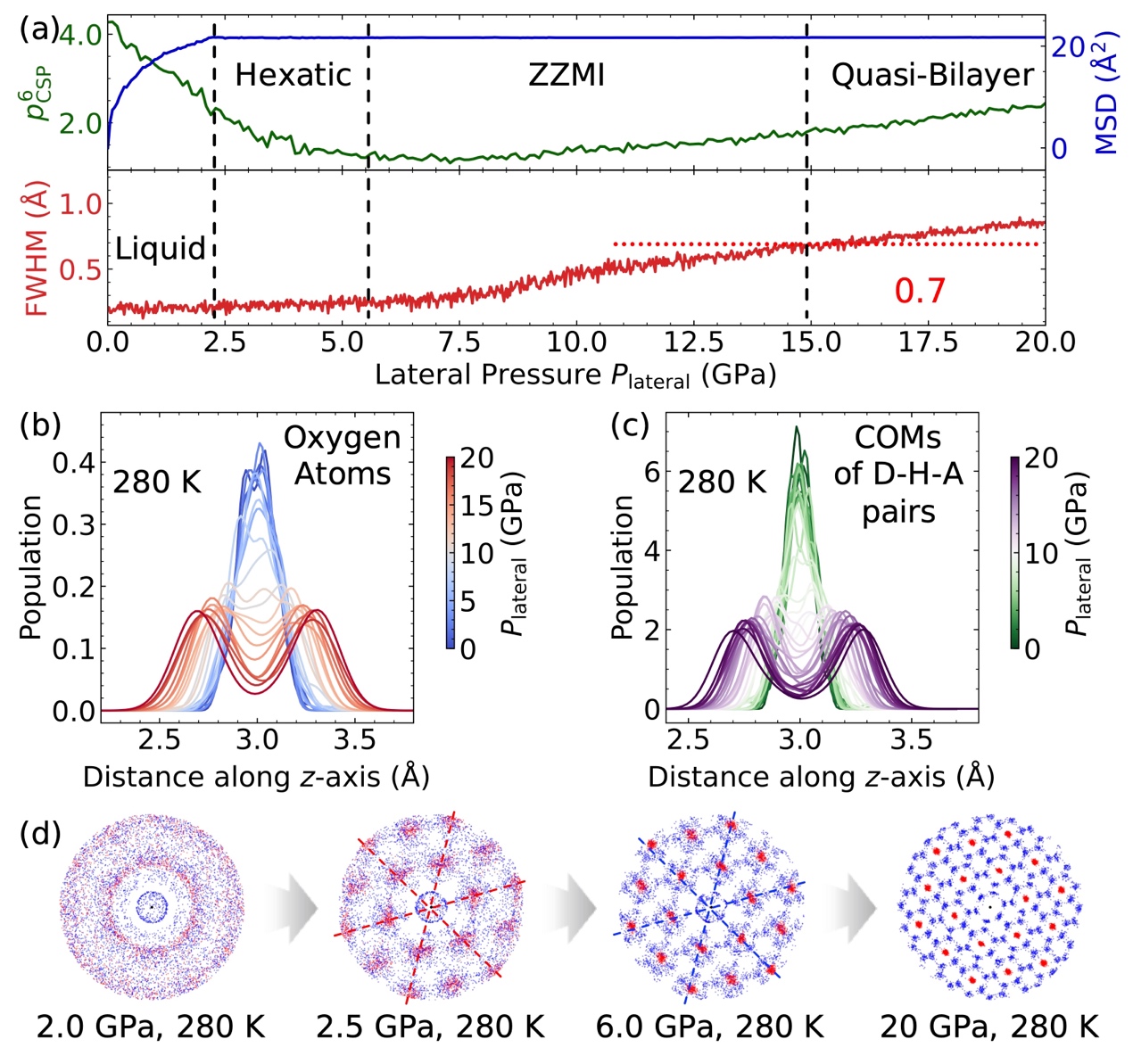


**Supplementary Fig. 11** Molecular dynamics (MD) simulations of sequential phase transition of the 2D water at 280 K. (a) Variation of centro-symmetry parameter (CSP) on six nearest oxygen atoms ($\text{p}_{\text{CSP}}^{\text{6}}$, green curves), mean squared displacement (MSD, blue curves) and full width at half maximum (FWHM, red curves) of the distribution function defined in (b). (c) The distribution functions of center-of-mass (COM) of donor-hydrogen-acceptor (D-H-A) pairs along the *z*-axis evolve with the lateral pressure. (d) Spatial distributions of oxygen (red dots) and hydrogen atoms (blue dots) within a cutoff of 6 Å at different time slots of the MD simulation. ZZMI is the abbreviation for zigzag monolayer ice.


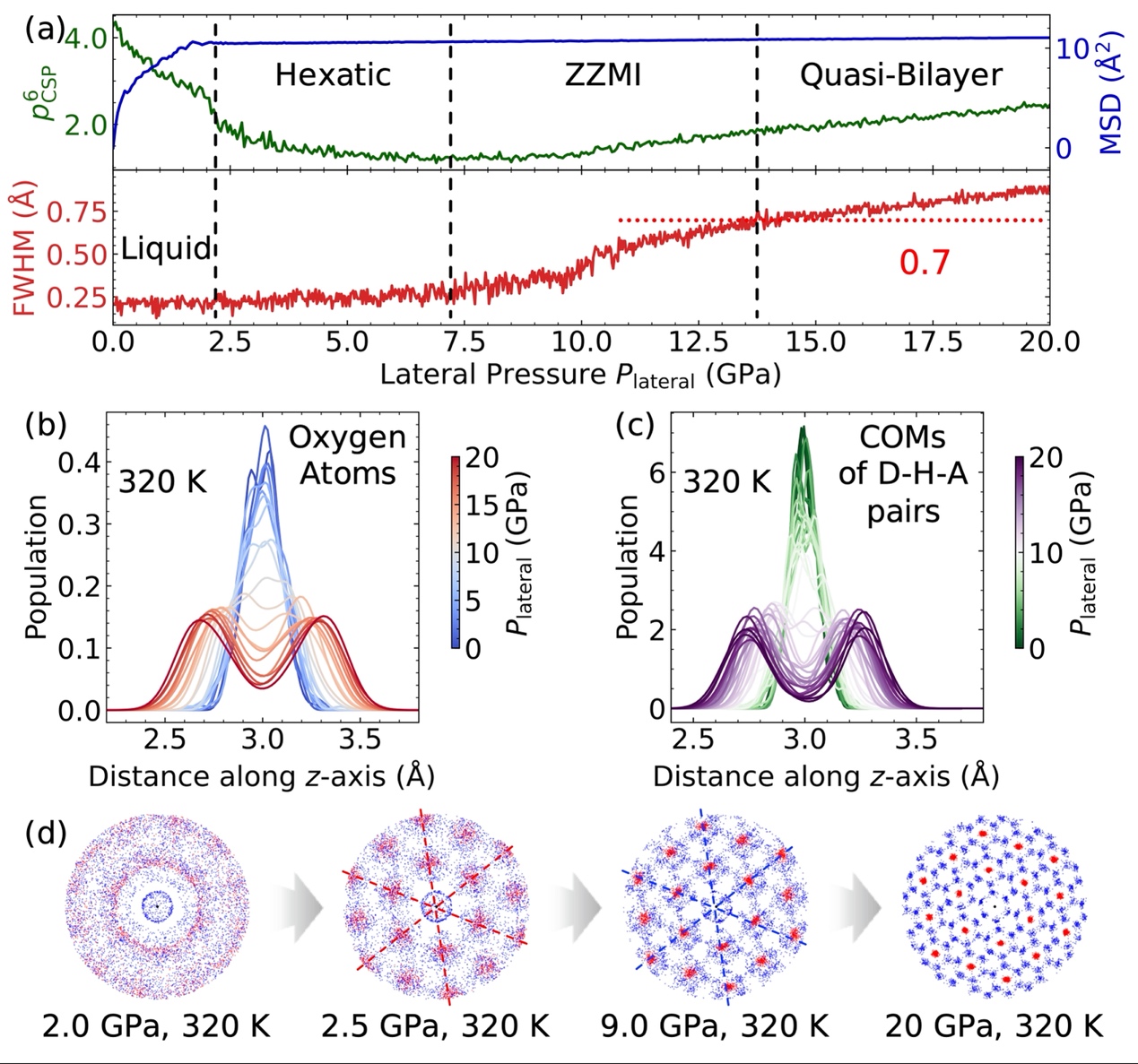


**Supplementary Fig. 12** Molecular dynamics (MD) simulations of sequential phase transition of the 2D water at 320 K. (a) Variation of centro-symmetry parameter on six nearest oxygen atoms ($\text{p}_{\text{CSP}}^{\text{6}}$, green curves), mean squared displacement (MSD, blue curves) and full width at half maximum (FWHM, red curves) of the distribution function defined in (b). (c) The distribution functions of center-of-mass (COM) of donor-hydrogen-acceptor (D-H-A) pairs along the *z*-axis evolve with the lateral pressure. (d) Spatial distributions of oxygen (red dots) and hydrogen atoms (blue dots) within a cutoff of 6 Å at different time slots of the MD simulation. ZZMI is the abbreviation for zigzag monolayer ice.


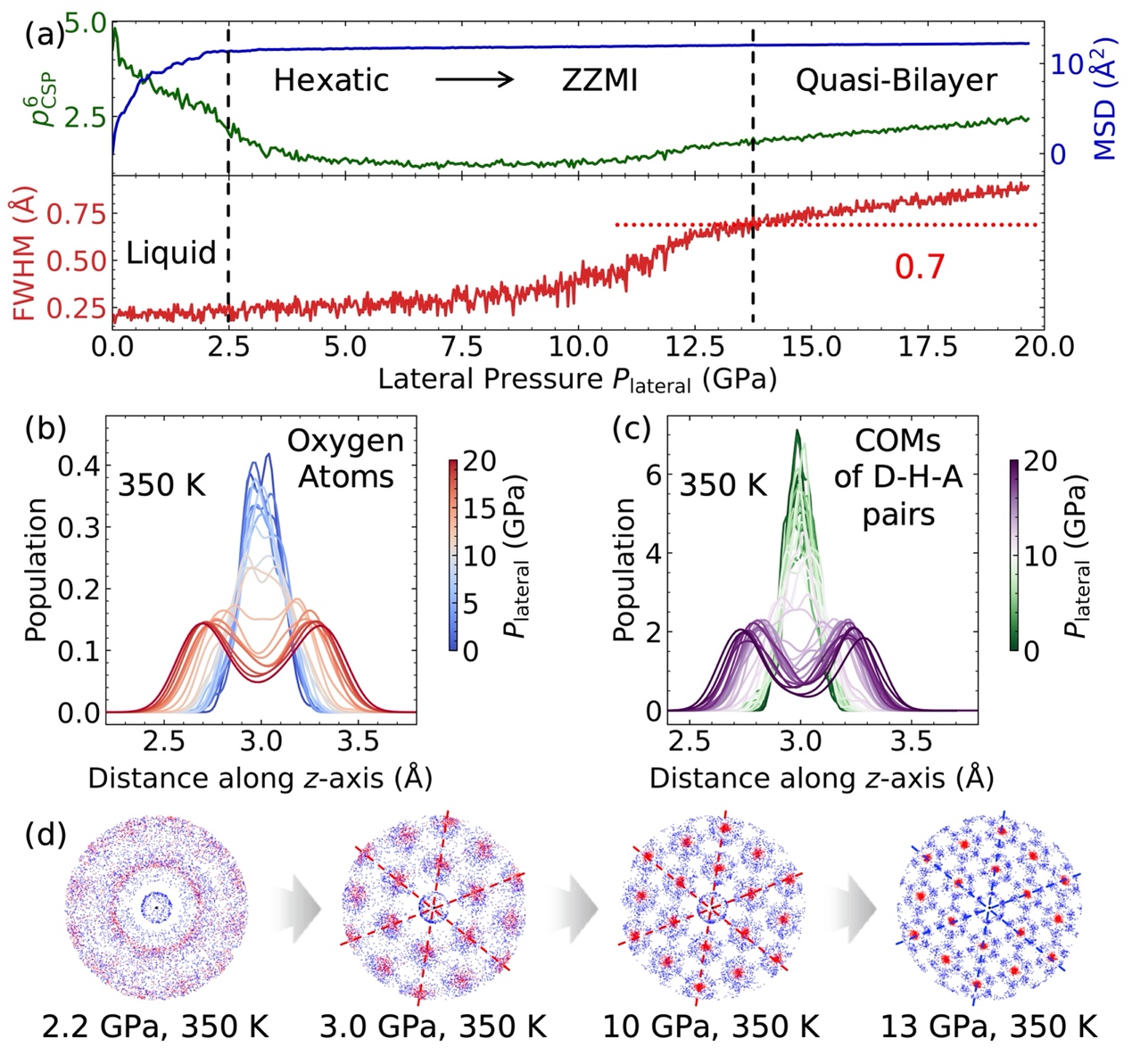


**Supplementary Fig. 13** Molecular dynamics (MD) simulations of sequential phase transition of the 2D water at 350 K. (a) Variation of centro-symmetry parameter on six nearest oxygen atoms ($\text{p}_{\text{CSP}}^{\text{6}}$, green curves), mean squared displacement (MSD, blue curves) and full width at half maximum (FWHM, red curves) of the distribution function defined in (b). (c) The distribution functions of center-of-mass (COM) of donor-hydrogen-acceptor (D-H-A) pairs along the *z*-axis evolve with the lateral pressure. (d) Spatial distributions of oxygen (red dots) and hydrogen atoms (blue dots) within a cutoff of 6 Å at different time slots of the MD simulation. ZZMI is the abbreviation for zigzag monolayer ice.


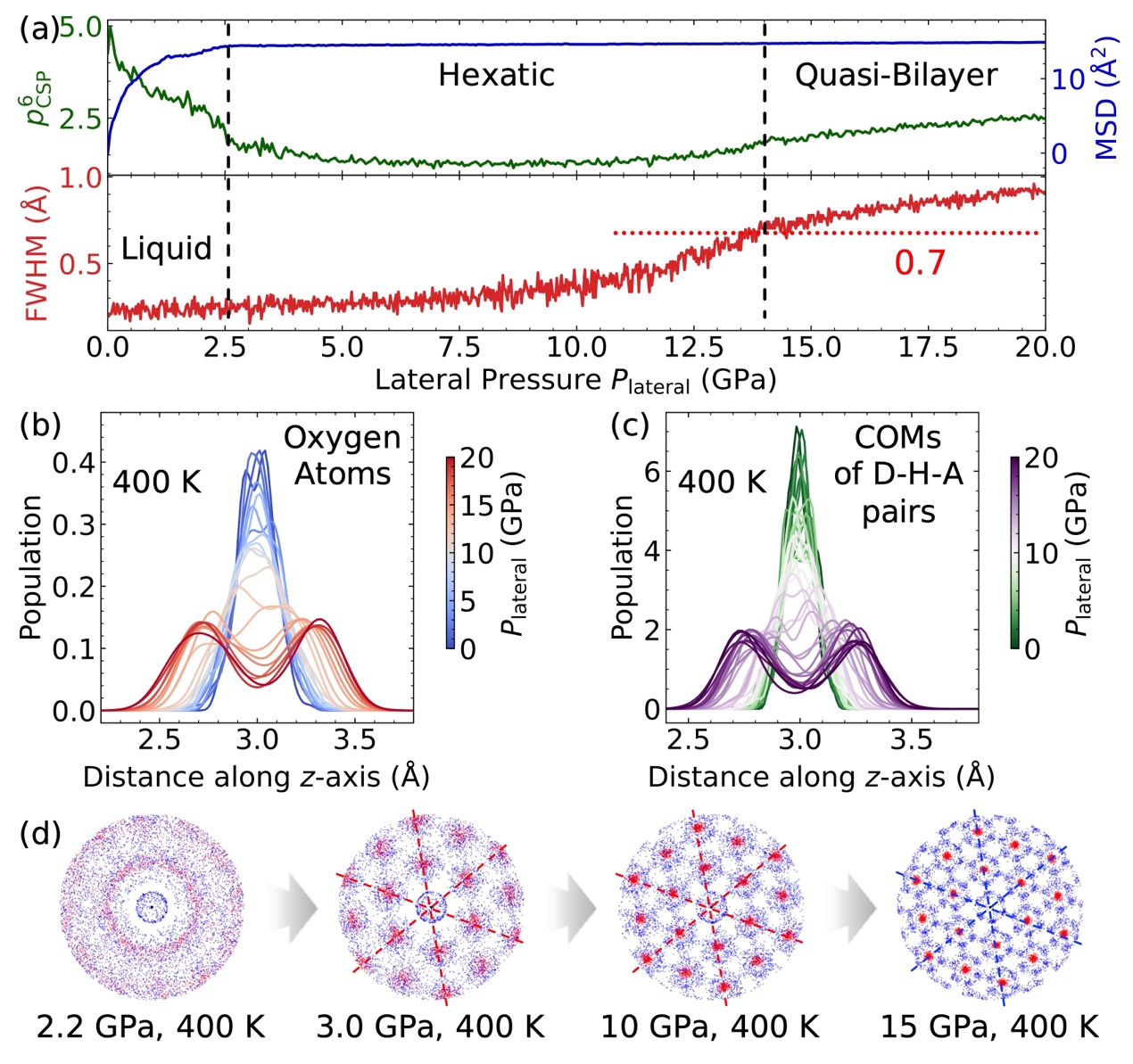


**Supplementary Fig. 14** Molecular dynamics (MD) simulations of sequential phase transition of the 2D water at 400 K. (a) Variation of centro-symmetry parameter on six nearest oxygen atoms ($\text{p}_{\text{CSP}}^{\text{6}}$, green curves), mean squared displacement (MSD, blue curves) and full width at half maximum (FWHM, red curves) of the distribution function defined in (b). (c) The distribution functions of center-of-mass (COM) of donor-hydrogen-acceptor (D-H-A) pairs along the *z*-axis evolve with the lateral pressure. (d) Spatial distributions of oxygen (red dots) and hydrogen atoms (blue dots) within a cutoff of 6 Å at different time slots of the MD simulation.


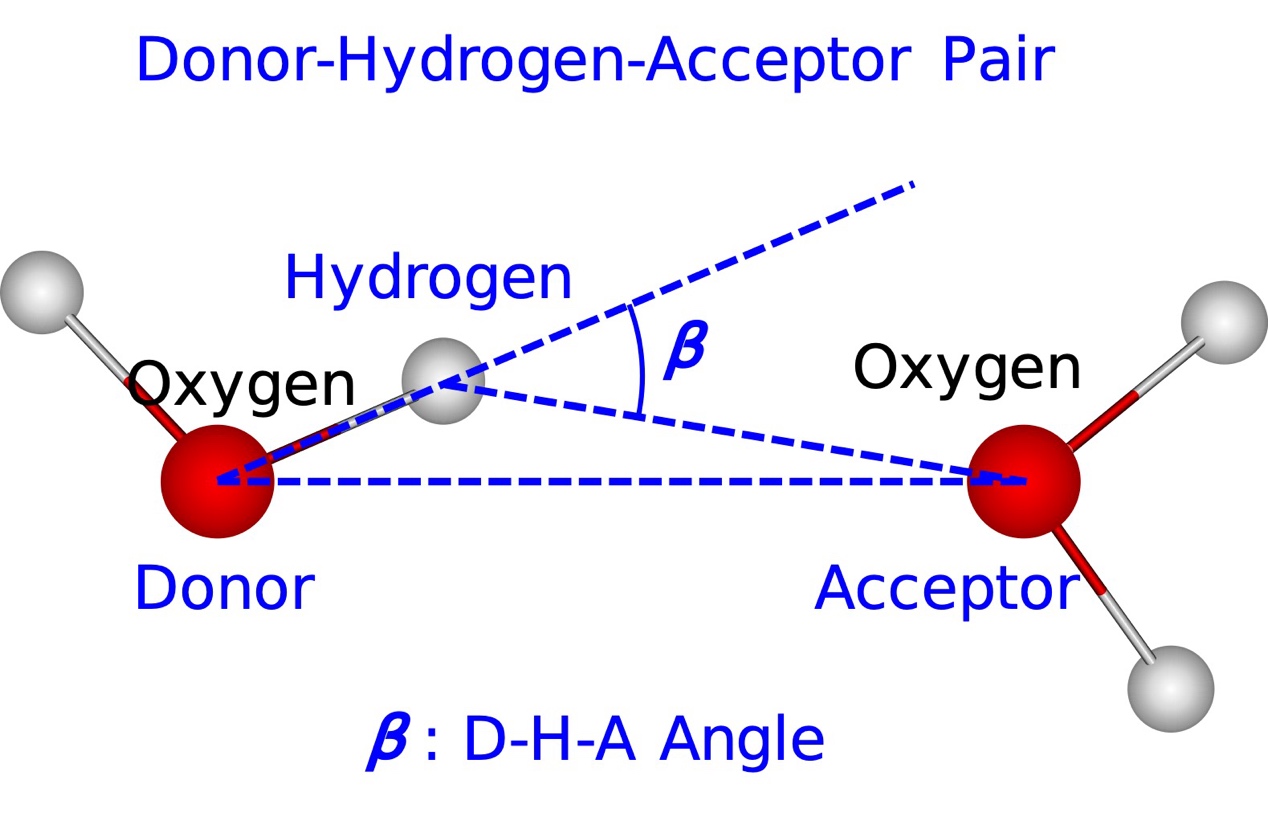


**Supplementary Fig. 15** A schematic illustration of the donor-hydrogen-acceptor (D-H-A) angle. The same color code as in Fig. 2 is used.


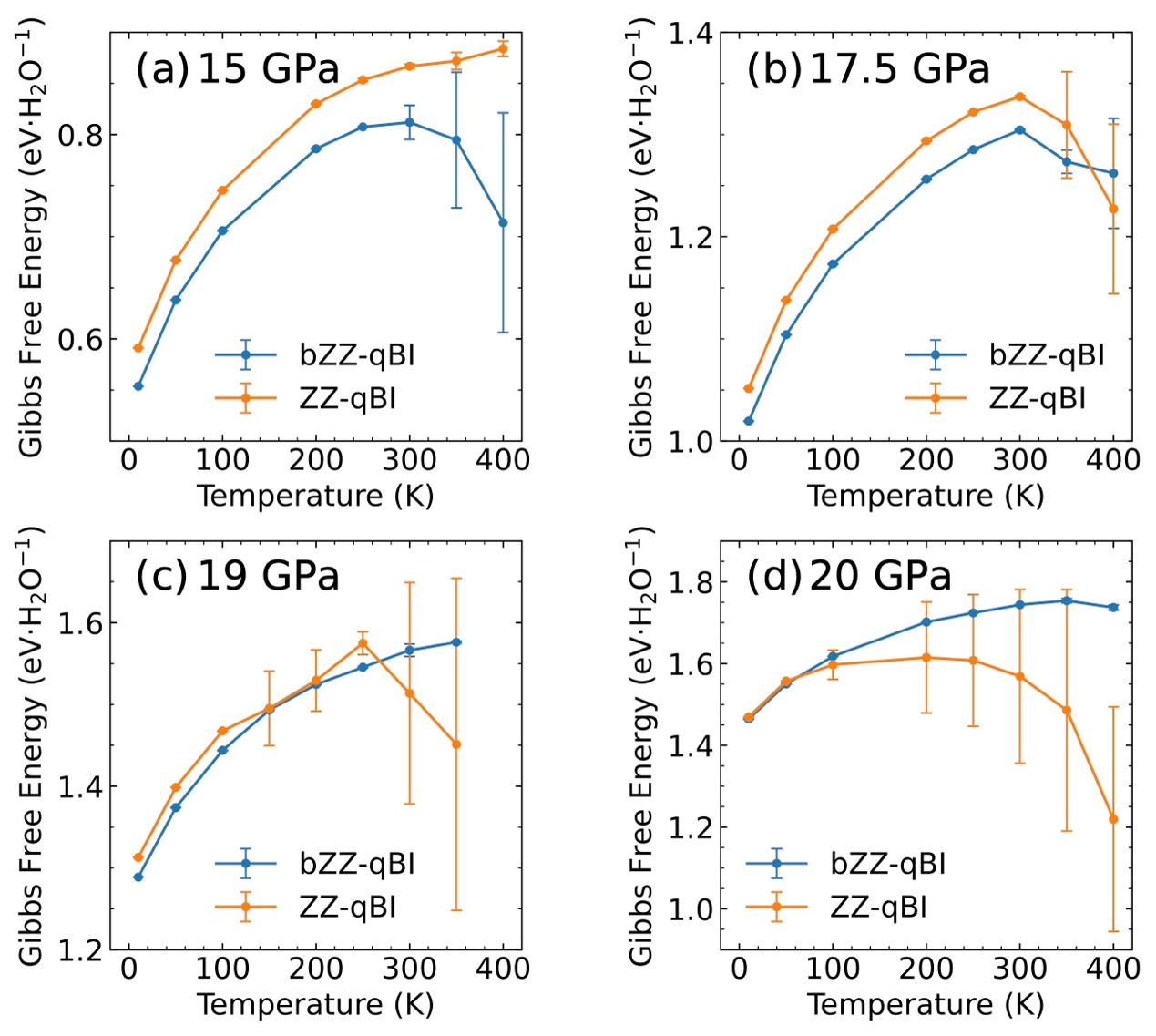


**Supplementary Fig. 16** Variation of Gibbs free energy of zigzag quasi-bilayer ice (ZZ-qBI) and branched-ZZ-qBI (bZZ-qBI) with temperature at various pressures. (a) 15 GPa, (b) 17.5 GPa, (c) 19 GPa and (d) 20 GPa. The error bar represents the uncertainty of the calculated Gibbs free energy, and the computational details can be found in the method section of “Determination of phase boundaries” in the main text.


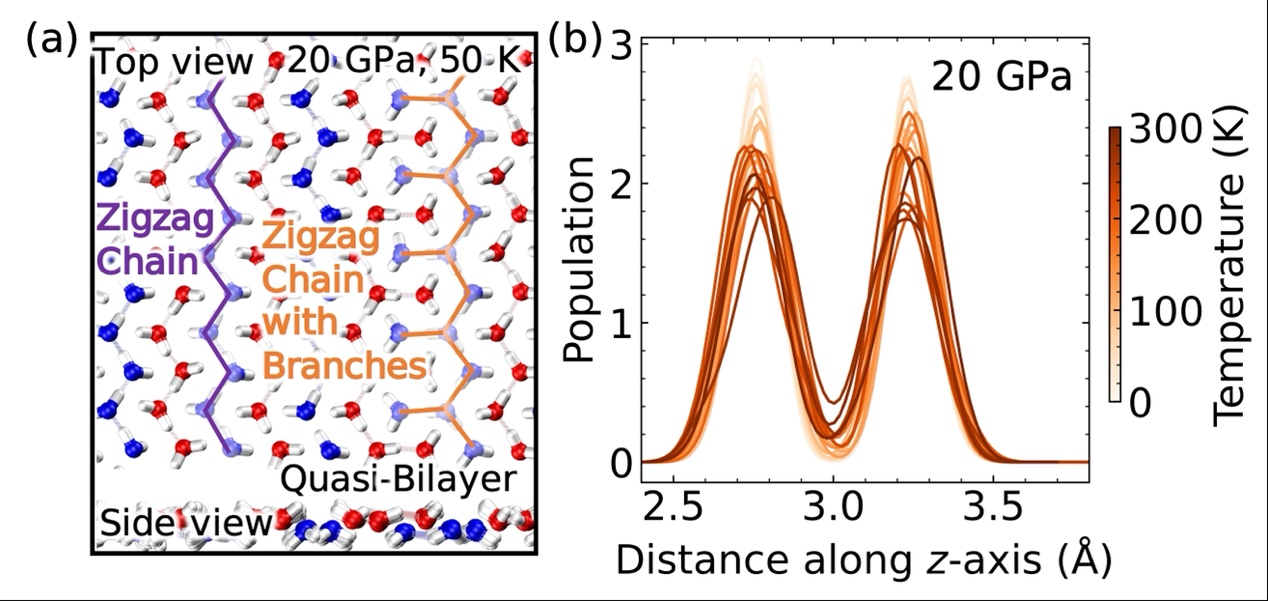


**Supplementary Fig. 17** Partial formation of quasi-bilayer at 20 GPa. (a) Atomic visualization of the partial formation of a quasi-bilayer ice directly transformed from a flat zigzag monolayer ice (ZZMI) at 20 GPa and 50 K. Red and blue spheres respectively represent oxygen atoms at top and bottom sublayers, and the corresponding hydrogen bonds are respectively red and blue dashed lines, and hydrogen atoms and oxygen-hydrogen (O-H) bonds are respectively white spheres and rods. (b) The distribution functions of center-of-mass (COM) of donor-hydrogen-acceptor (D-H-A) pairs along the *z*-axis evolve with the temperature at 20 GPa.


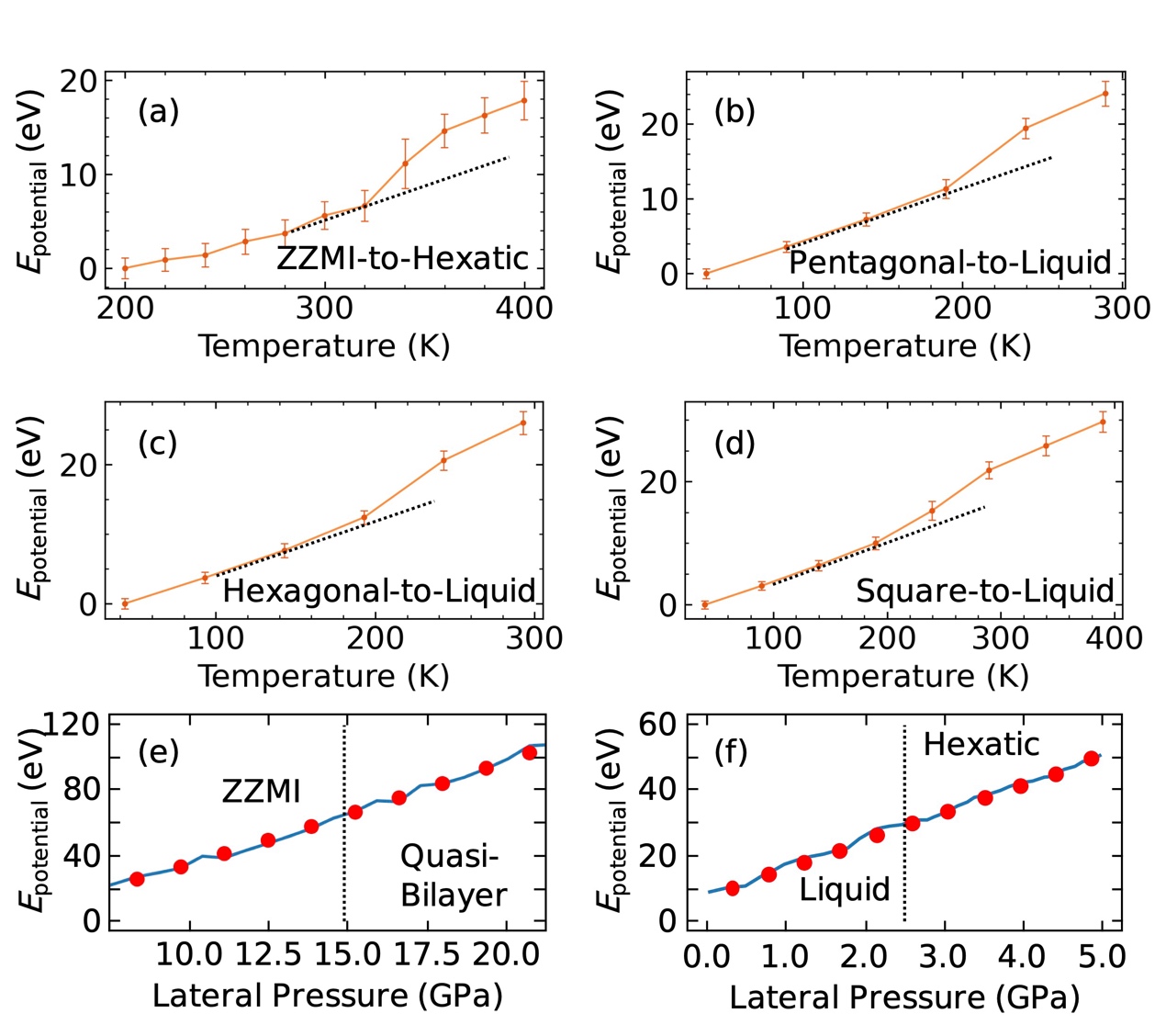


**Supplementary Fig. 18** Potential energy (*E*_potential_) variation of the 2D monolayer ice/water during phase transition. (a) ZZMI-to-hexatic, (b) pentagonal-to-liquid, (c) hexagonal-to-liquid and (d) square-to-liquid transitions, (e) ZZMI-to-bZZ-qBI and (f) liquid-to-hexatic transitions. Red dots were averaged every 10000 steps. ZZMI and bZZ-qBI represent zigzag monolayer ice and branched zigzag quasi-bilayer ice, respectively. The error bar represents the uncertainty of the average value for ~1000 instant records in molecular dynamics simulations.


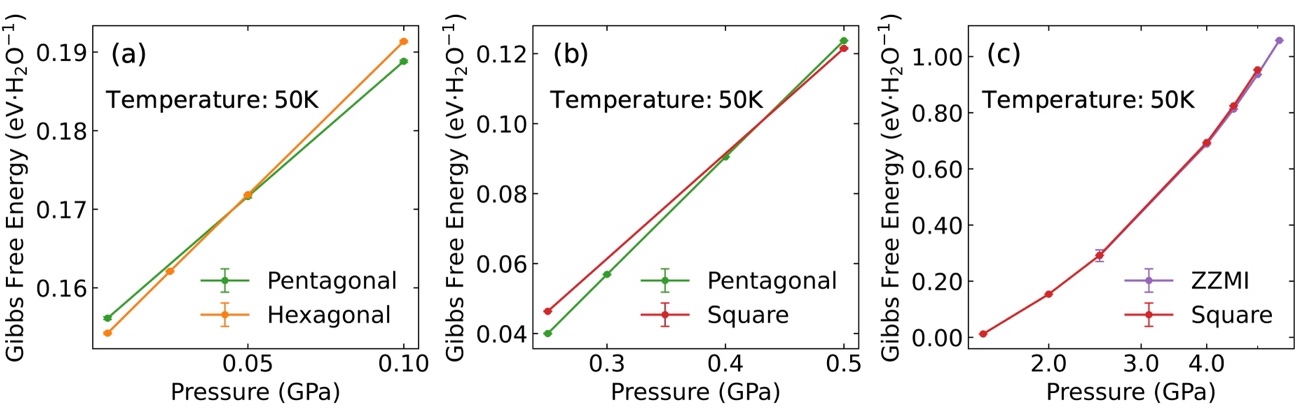


**Supplementary Fig. 19** The variation of Gibbs free energy with lateral pressure during phase transitions. (a) pentagonal-to-hexagonal, (b) pentagonal-to-square, and (c) square-to-ZZMI (zigzag quasi-bilayer ice) transition. The error bar represents the uncertainty of the calculated Gibbs free energy, and the computational details can be found in the method section of “Determination of phase boundaries” in the main text.


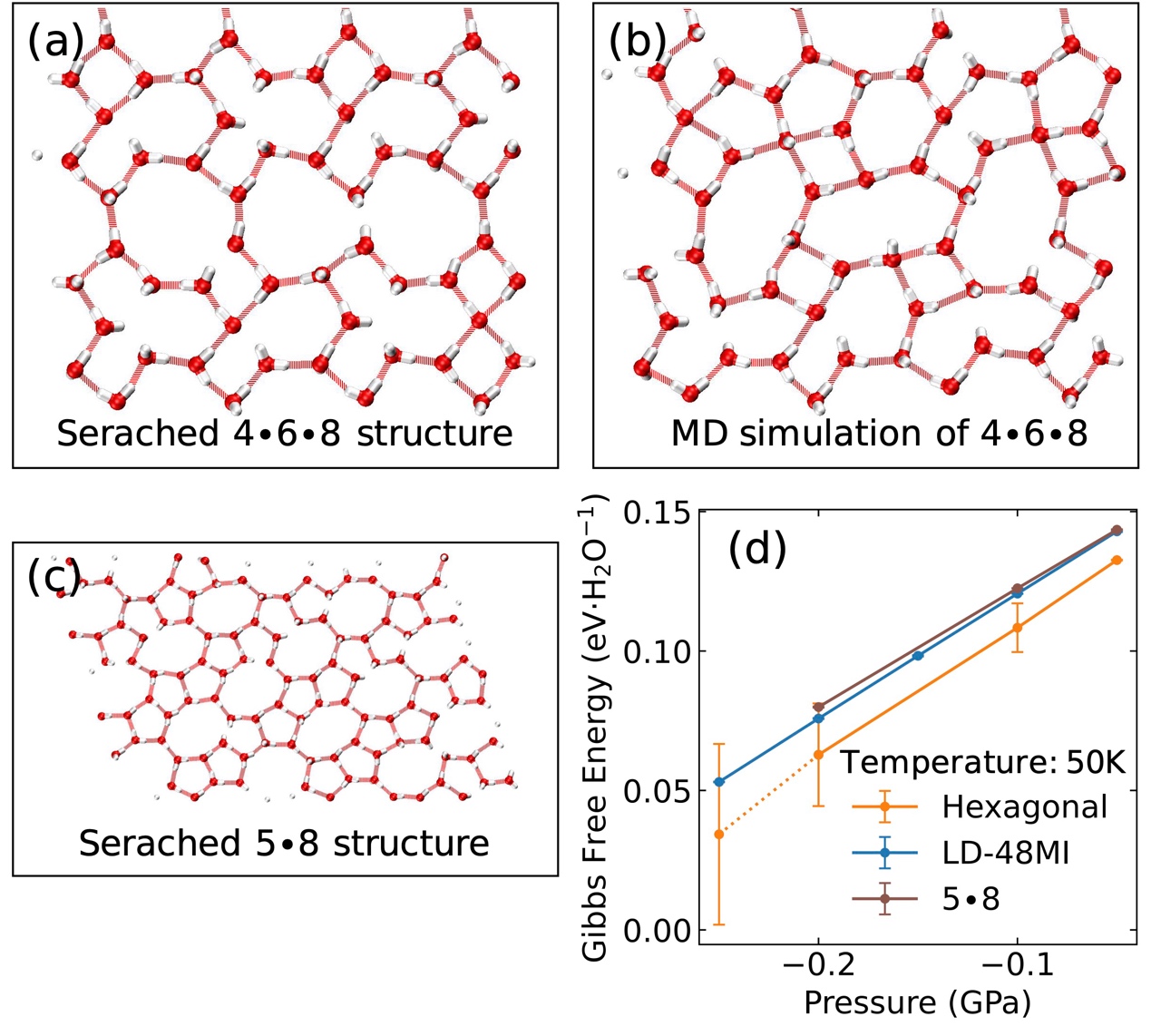


**Supplementary Fig. 20** Ultra low-density phases observed in our simulations. (a) Structure of the 2D 4∙6∙8 ice obtained from using the random structure search method, (b) Snapshot of the molecular dynamics (MD) simulation of the 4∙6∙8 ice based on the machine-learning force field (MLFF) model. (c) Structure of the 2D 5∙8 ice from using the random structure search method, (d) Variation of Gibbs free energy with pressure for the hexagonal (orange), 5∙8 ice (brown) and the low-density 4⋅8^2^ monolayer ice (LD-48MI, blue line) at 50 K. The error bar represents the uncertainty of the calculated Gibbs free energy, and the computational details can be found in the method section of “Determination of phase boundaries” in the main text.


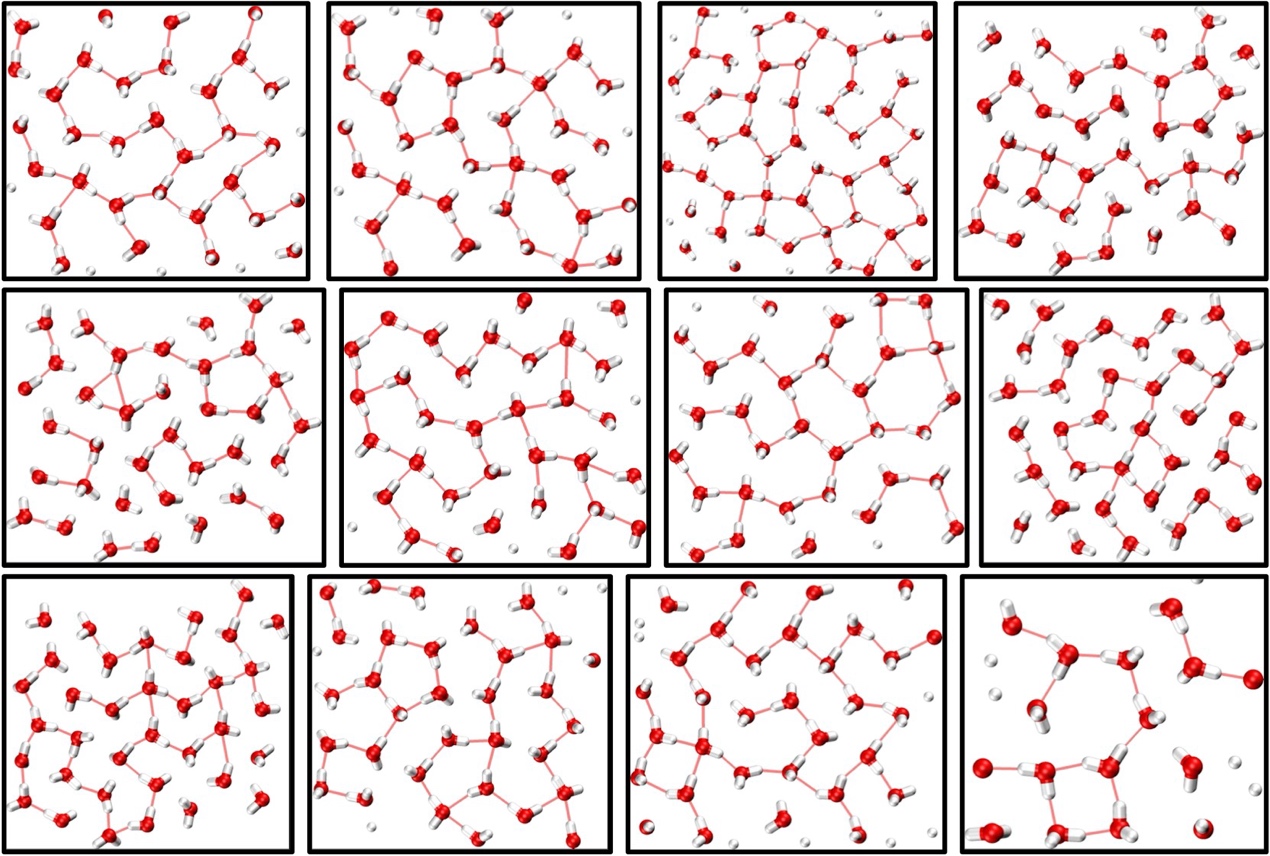


**Supplementary Fig. 21** Representative atomic images of the liquid in the training data. White and red spheres are hydrogen and oxygen atoms, respectively.


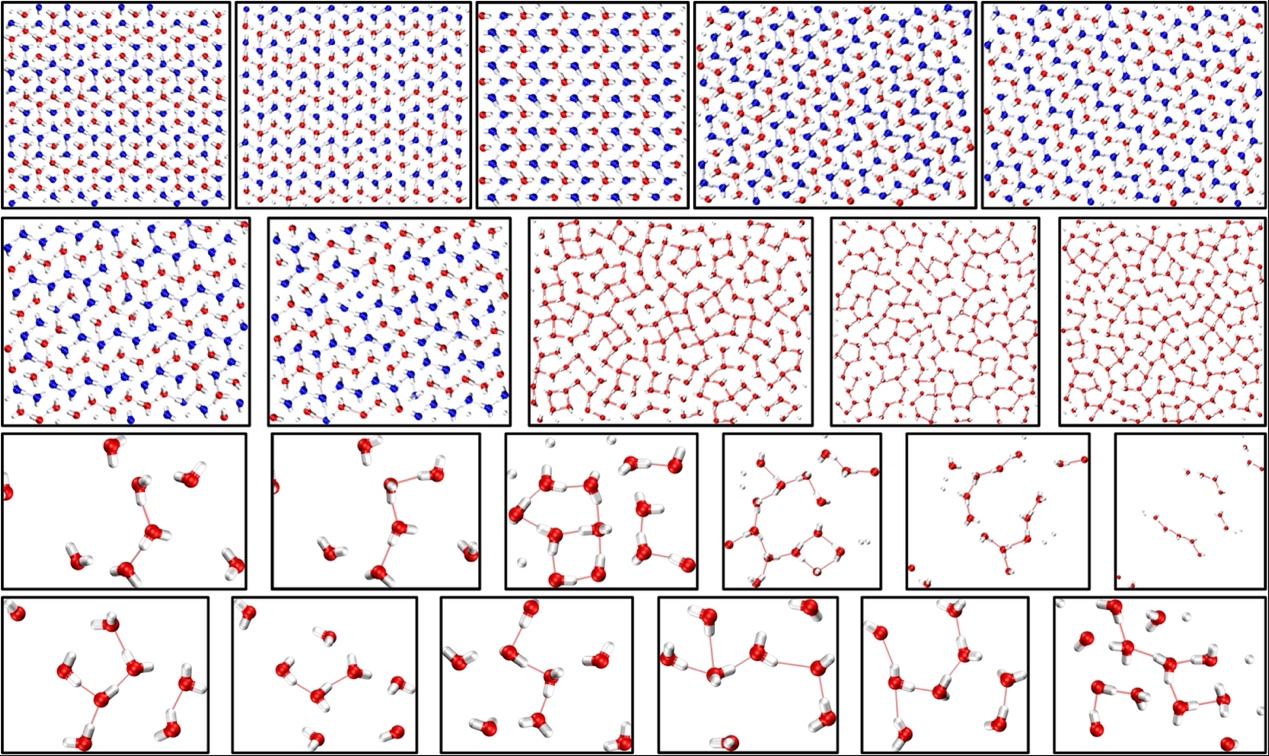


**Supplementary Fig. 22** Representative atomic images in the validation data. White and red spheres are hydrogen and oxygen atoms, respectively. Blue and red spheres in the first seven images are oxygen atoms on the upper and lower sublayer of the new quasi-bilayer phases.


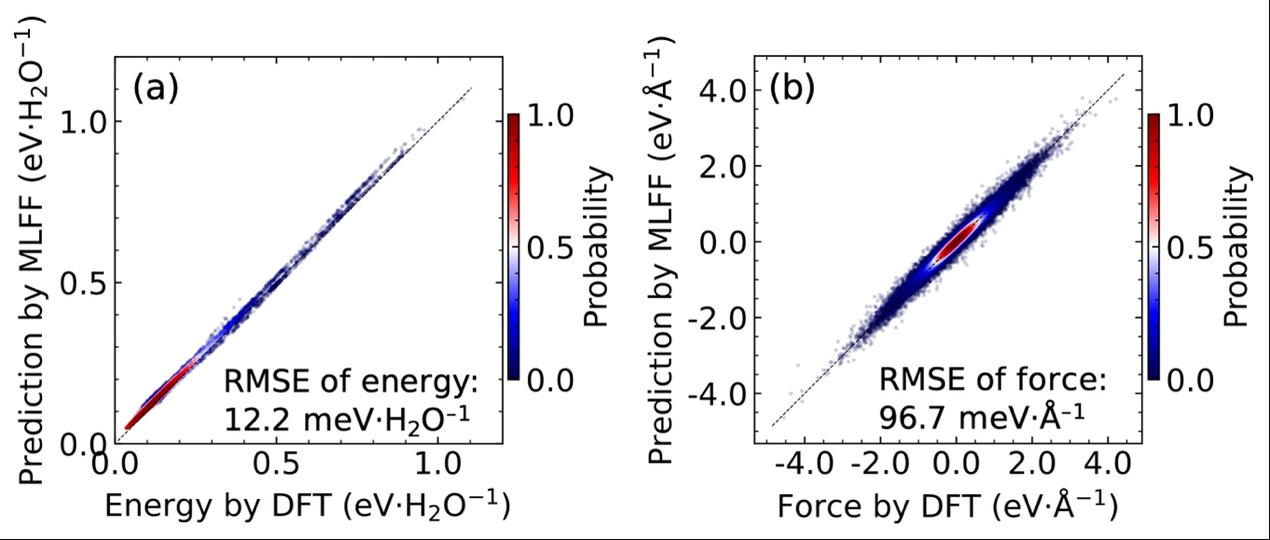


**Supplementary Fig. 23** Performance of the machine-learning force field (MLFF) model on the validation data. (a) The prediction-true plots of energy and (b) atomic forces. The color represents the probability distribution of the “true-prediction” points. The probability decreases from red to blue. The validation data contain 11,217 atomic images from MLFF molecular dynamics (MD) and ab initio MD (AIMD) simulations. DFT and RMSE are the abbreviations for density functional theory and root-mean-square error, respectively.


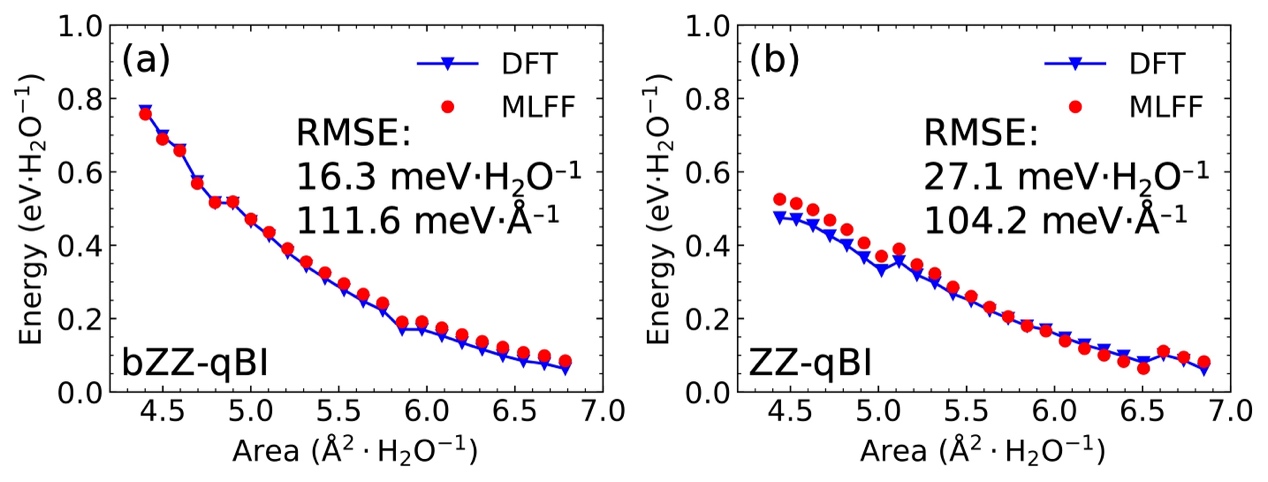


**Supplementary Fig. 24** Energy-area (*E-A*) curves obtained from the density functional theory (DFT) calculations and the machine-learning force field (MLFF) model. (a) Branched zigzag quasi-bilayer ice (bZZ-qBI). (b) zigzag quasi-bilayer ice (ZZ-qBI). Root mean square errors (RMSE) of the energy (in meV⋅H_2_O^−1^) and forces (meV⋅Å^−1^) are shown by the curve.


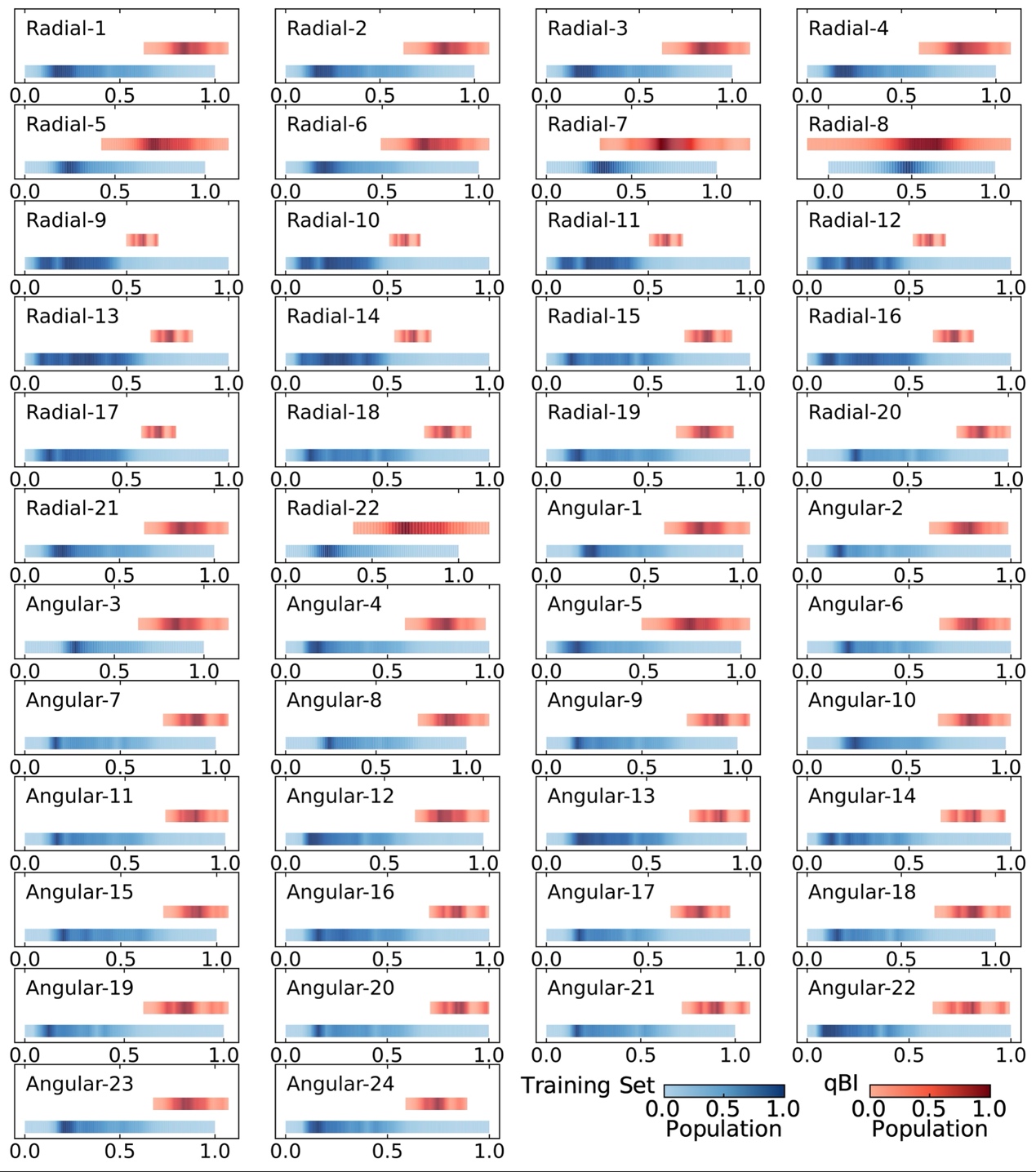


**Supplementary Fig. 25** Probability distribution of 46 O-centered Behler-Parrinello symmetry functions. The red and blue bars are for the quasi-bilayer (qBI) and atomic images in training data, respectively. The symmetry functions were indexed in the appearance order shown in Ref. 48.


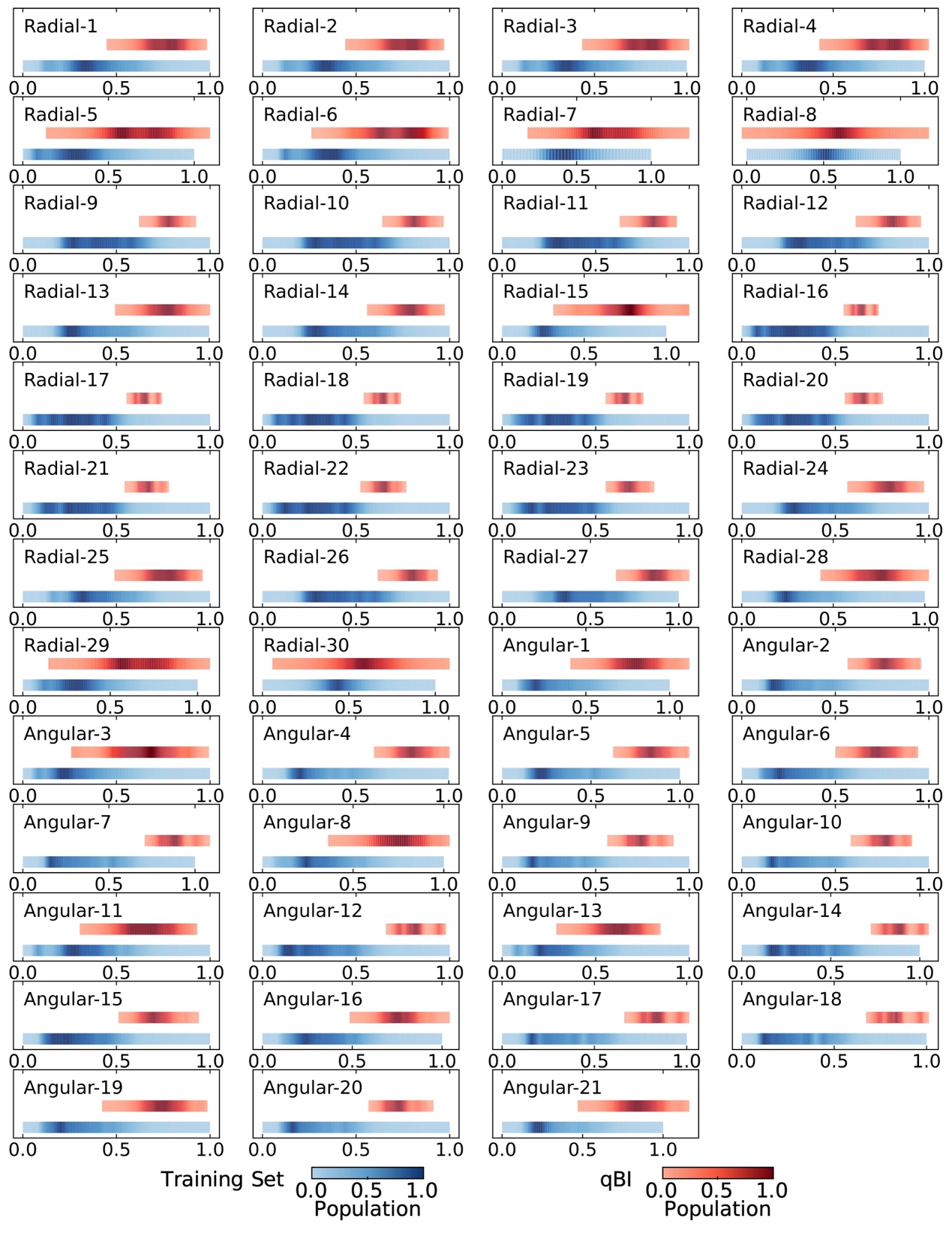


**Supplementary Fig. 26** Probability distribution of 51 H-centered Behler-Parrinello symmetry functions. The red and blue bars are for the quasi-bilayer (qBI) and atomic images in training data, respectively. The symmetry functions were indexed in the appearance order shown in Ref. 48.


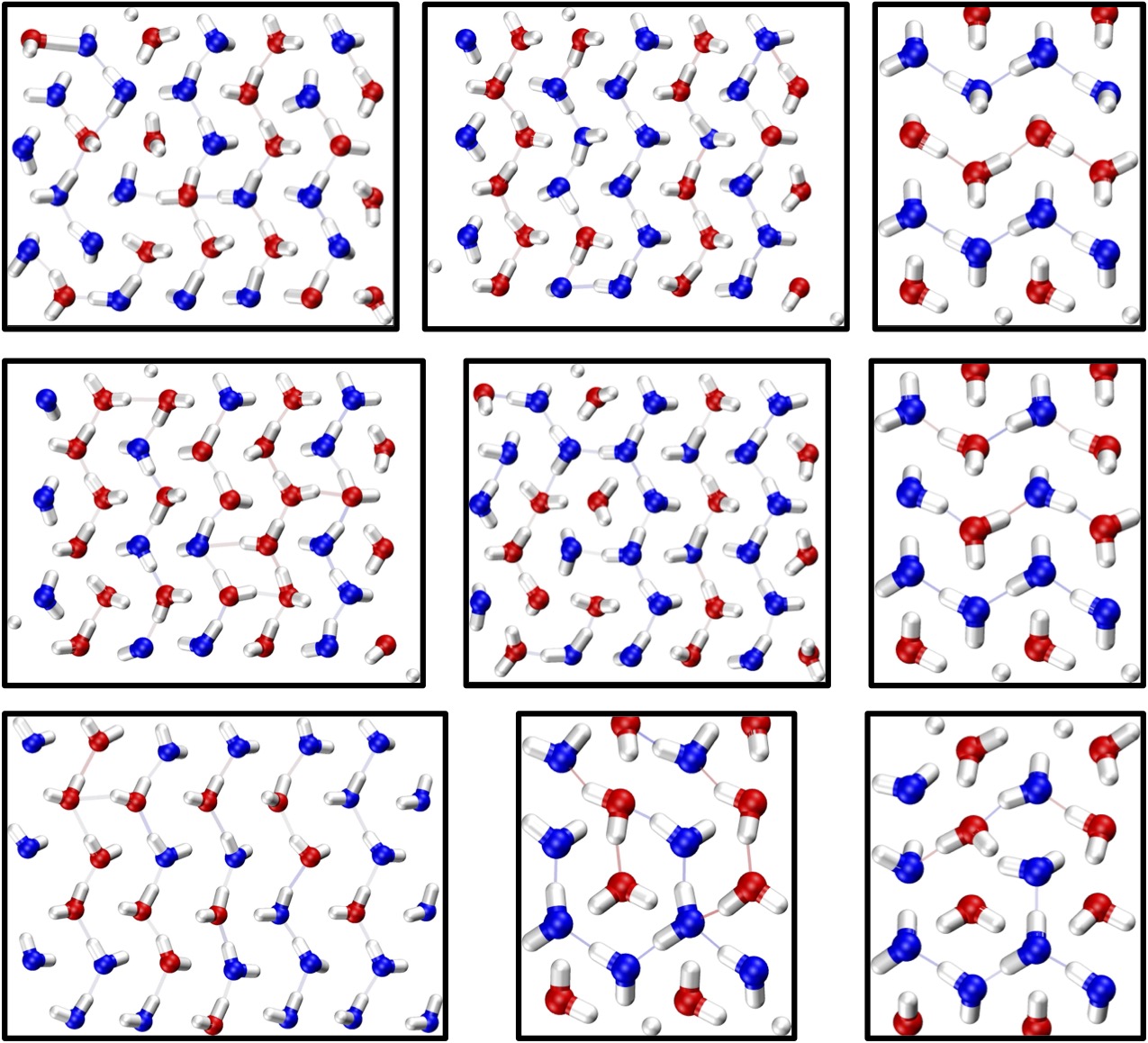


**Supplementary Fig. 27** Representative atomic images in the training data. The shown atomic images have local environments of the zigzag quasi-bilayer ice (ZZ-qBI) and branched-ZZ-qBI (bZZ-qBI). White spheres are hydrogen atoms. Blue and red spheres in the first seven images are oxygen atoms on the upper and lower sublayer of the new quasi-bilayer phases.


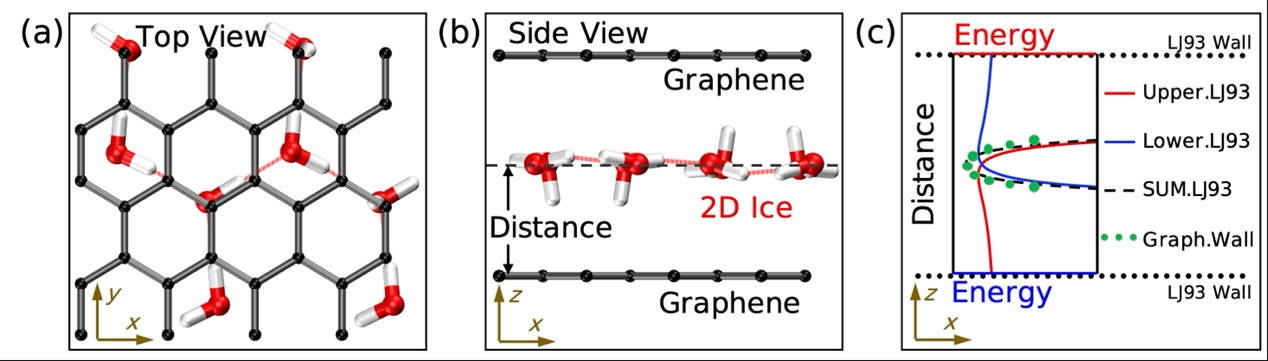


**Supplementary Fig. 28** Comparison of the realistic graphene wall and the LJ93 wall. (a) Top and (b) side view of a 2D ice confined between two graphene monolayers. (c) A nanoslit model constructed with the LJ93 smooth walls that reproduces the nanoslit with realistic graphene walls shown in (a) and (b).
