# Supplementary material for: Temperature-pressure phase diagram of confined monolayer water/ice at first-principles accuracy with a machine-learning force field
Source: Nat Commun. 2023 Jul 11;14:4110. doi: 10.1038/s41467-023-39829-z (PMC10336112; doi:10.1038/s41467-023-39829-z)
Supplement: Supplementary file 2 — Description of Additional Supplementary Files [file 41467_2023_39829_MOESM2_ESM.pdf]

File name: Supplementary Movie 1
Description: An MLFF MD simulation of spontaneous formation of the hexagonal monolayer ice from the 2D water.

File name: Supplementary Movie 2
Description: An MLFF MD simulation of spontaneous formation of the pentagonal monolayer ice from the 2D water.

File name: Supplementary Movie 3
Description: An MLFF MD simulation of spontaneous formation of the square monolayer ice by MLFF MD simulations.

File name: Supplementary Movie 4
Description: An MLFF MD simulation of spontaneous formation of the ZZMI monolayer ice by MLFF MD simulations.

File name: Supplementary Movie 5
Description: An MLFF MD simulation of spontaneous hexagonal-to-pentagonal transition.

File name: Supplementary Movie 6
Description: An MLFF MD simulation of spontaneous pentagonal-to-square transition.

File name: Supplementary Movie 7
Description: An MLFF MD simulation of spontaneous square-to-ZZMI transition.

File name: Supplementary Movie 8
Description: An MLFF MD simulation of spontaneous square-to-pentagonal transition.

File name: Supplementary Movie 9
Description: An MLFF MD simulation of phase transition from the 2D water to a final ZZ-qBI at 300 K with lateral pressure increasing from 0 to 20 GPa.

File name: Supplementary Movie 10
Description: An MLFF MD simulation of branched ZZ-qBI at constant lateral pressure of 15 GPa with temperature increasing from 10 K to 300 K.

File name: Supplementary Movie 11
Description: A 27-ps AIMD simulation of a ZZ-qBI ice at 20 GPa and 300 K.

File name: Supplementary Movie 12
Description: A 35-ps AIMD simulation of a bZZ-qBI ice at 15 GPa and 300 K.

File name: Supplementary Movie 13
Description: A 21-ps rev-PBE0-D3 AIMD simulation of a ZZ-qBI ice at 20 GPa and 300 K.

File name: Supplementary Movie 14
Description: A 26-ps rev-PBE0-D3 AIMD simulation of a bZZ-qBI ice at 15 GPa and 300 K.

File name: Supplementary Movie 15
Description: A 27-ps AIMD simulation of a ZZ-qBI ice at 300 K with real graphene walls.

File name: Supplementary Movie 16
Description: A 23-ps AIMD simulation of a bZZ-qBI ice at 300 K with real graphene walls.

File name: Supplementary Movie 17
Description: A 20-ps AIMD simulation of a ZZ-qBI ice at 350 K with real graphene walls.

File name: Supplementary Movie 18
Description: A 20-ps AIMD simulation of a bZZ-qBI ice at 350 K with real graphene walls.

File name: Supplementary Movie 19
Description: A 20-ps BLYP-D3 AIMD simulation of a ZZ-qBI ice at 350 K with real graphene walls.

File name: Supplementary Movie 20
Description: A 20-ps BLYP-D3 AIMD simulation of a bZZ-qBI ice at 350 K with real graphene walls.

File name: Supplementary Movie 21
Description: A 11-ps revPBE0-D3 AIMD simulation of a ZZ-qBI ice at 350 K with real graphene walls.

File name: Supplementary Movie 22
Description: A 1-ns PIMD simulation of a ZZ-qBI ice at 300 K, using a timestep of 0.25 fs.

File name: Supplementary Movie 23
Description: A 1-ns PIMD simulation of a bZZ-qBI ice at 300 K, using a timestep of 0.25 fs.

File name: Supplementary Movie 24
Description: A 1-ns MLFF MD simulation of ZZ-qBI at 300 K and 8-10 GPa within a nanoslit of 6.5 Å width, using a timestep of 0.2 fs.

File name: Supplementary Movie 25
Description: A 1-ns MLFF MD simulation of bZZ-qBI at 300 K and 10-15 GPa within a nanoslit of 6.5 Å width, using a timestep of 0.2 fs.

File name: Supplementary Movie 26
Description: A 1-ns MLFF MD simulation of ZZ-qBI at 300 K and >50 GPa within a nanoslit of 5.5 Å width, using a timestep of 0.2 fs.

File name: Supplementary Movie 27
Description: A 1-ns MLFF MD simulation of bZZ-qBI at 300 K and >50 GPa within a nanoslit of 5.5 Åwidth, using a timestep of 0.2 fs.

File name: Supplementary Movie 28
Description: A 1-ns PIMD simulation of transformation of qBI to ZZMI at 300 K within a nanoslit of 5.0 Å width, using a timestep of 0.25 fs.

File name: Supplementary Movie 29
Description: A 26-ps AIMD simulation of a hexagonal ice at 0.025 GPa and 150 K.

File name: Supplementary Movie 30
Description: A 20-ps AIMD simulation of a pentagonal ice at 0.25 GPa and 150 K with real graphene walls.

File name: Supplementary Movie 31
Description: A 26-ps AIMD simulation of a square ice at 2.5 GPa and 200 K.

File name: Supplementary Movie 32
Description: A 26-ps AIMD simulation of a ZZMI ice at 10.0 GPa and 300 K.

File name: Supplementary Movie 33
Description: A 21-ps AIMD simulation of the LD-48MI at -0.4 GPa and 50 K with real graphene walls.

File name: Supplementary Movie 34
Description: A 2.9-ps AIMD simulation of spontaneous LD-48MI-to-gas transition at -0.5 GPa and 100 K.

File name: Supplementary Movie 35
Description: A 11.1-ps AIMD simulation of spontaneous LD-48MI-to-gas transition at -0.3 GPa and 150 K.

File name: Supplementary Movie 36
Description: A 3.4-ps AIMD simulation of spontaneous hexagonal-to-gas transition at -0.25 GPa and 180 K.
